# Stochastic Signatures of Involuntary Head Micro-movements Can Be Used to Classify Females of ABIDE into Different Subtypes of Neurodevelopmental Disorders

**DOI:** 10.3389/fnint.2017.00010

**Published:** 2017-06-07

**Authors:** Elizabeth B. Torres, Sejal Mistry, Carla Caballero, Caroline P. Whyatt

**Affiliations:** ^1^Department of Psychology, Rutgers UniversityPiscataway, NJ, United States; ^2^Computer Science Department and Rutgers Center for Cognitive Science, Center for Biomedical Imaging and ModelingNew Brunswick, NJ, United States; ^3^Department of Biomathematics, Rutgers UniversityPiscataway, NJ, United States

**Keywords:** females, head micro-movements, autism, AS, stochastic signatures, resting state fMRI

## Abstract

**Background:** The approximate 5:1 male to female ratio in clinical detection of Autism Spectrum Disorder (ASD) prevents research from characterizing the female phenotype. Current open access repositories [such as those in the Autism Brain Imaging Data Exchange (ABIDE I-II)] contain large numbers of females to help begin providing a new characterization of females on the autistic spectrum. Here we introduce new methods to integrate data in a scale-free manner from continuous biophysical rhythms of the nervous systems and discrete (ordinal) observational scores.

**Methods:** New data-types derived from image-based involuntary head motions and personalized statistical platform were combined with a data-driven approach to unveil sub-groups within the female cohort. Further, to help refine the clinical DSM-based ASD vs. Asperger's Syndrome (AS) criteria, distributional analyses of ordinal score data from Autism Diagnostic Observation Schedule (ADOS)-based criteria were used on both the female and male phenotypes.

**Results:** Separate clusters were automatically uncovered in the female cohort corresponding to differential levels of severity. Specifically, the AS-subgroup emerged as the most severely affected with an excess level of noise and randomness in the involuntary head micro-movements. Extending the methods to characterize males of ABIDE revealed ASD-males to be more affected than AS-males. A thorough study of ADOS-2 and ADOS-G scores provided confounding results regarding the ASD vs. AS male comparison, whereby the ADOS-2 rendered the AS-phenotype worse off than the ASD-phenotype, while ADOS-G flipped the results. Females with AS scored higher on severity than ASD-females in all ADOS test versions and their scores provided evidence for significantly higher severity than males. However, the statistical landscapes underlying female and male scores appeared disparate. As such, further interpretation of the ADOS data seems problematic, rather suggesting the critical need to develop an entirely new metric to measure social behavior in females.

**Conclusions:** According to the outcome of objective, data-driven analyses and subjective clinical observation, these results support the proposition that the female phenotype is different. Consequently the “*social behavioral male ruler*” will continue to mask the female autistic phenotype. It is our proposition that new observational behavioral tests ought to contain normative scales, be statistically sound and combined with objective data-driven approaches to better characterize the females across the human lifespan.

## Introduction

Autism Spectrum Disorder (ASD) presents a diagnosis ratio estimated between 4:1 and 5:1 males to females (Volkmar et al., 1993; Mandy et al., 2012), a figure that is further exacerbated by evidence indicating that females are diagnosed significantly later than males (Lai et al., 2015). Indeed, studies show that observational clinical tools, such as the Diagnostic Statistical Manual (DSM) [ASD; APA 4] and Autism Diagnostic Observation Schedule (ADOS) (Lord et al., 2000, 2012) may need modifications to detect symptomatology earlier in females. Such adaptations could help further our understanding of differential sex contribution to the ASD phenotype. Namely, the DSM-V shows a marked division from the DSM IV by encompassing ASD, Asperger's Syndrome (AS), and other similar developmental disorders under an umbrella diagnostic label of Autism Spectrum Disorders, yet the diagnostic implications with respect to sex-level differences has yet to be elucidated. Unfortunately, the current diagnostic rates present tangible difficulties in exploring ASD within the wider female population—most notably challenges in recruiting a sufficient number of female participants. The current methods are therefore grounded on the observation of social behaviors within the male phenotype. However, we know that expectations of social behavior vary from culture to culture. As such, they carry a heavy subjective weight. Thus, the question posed is, how can we use objective means and take advantage of contemporary data-driven techniques, to assess the question of sex differences in ASD?

In recent years, access to open scientific repositories of data has enabled researchers to rethink the issue of sex differences in ASD—providing access to a range of data to achieve higher levels of statistical power and female representation. For instance, a number of publications have pointed at presumed, fundamental, differences in brain signal variability (Takahashi et al., 2016) and patterns of connectivity between the typically developing (TD) brain and the ASD brain (Cheng et al., 2015; Falahpour et al., 2016) by drawing on brain imaging data banks. Importantly, such research highlights specific sex-based differences (Alaerts et al., 2016), including differentiations in structural organization of the motor systems, which are discussed in light of repetitive behaviors (Supekar and Menon, 2015), cortical volume and gyrification (Schaer et al., 2015), among other morphological parameters. Such evidence for fundamental, physiological differences in ASD expression between the sexes may allude to new, refined methods to isolate and quickly identify ASD symptomatology in females; a population that has been thus far difficult to diagnose.

But how accurate and reliable are these claims? A series of recent papers have begun to question the “black-box” treatment of functional magnetic resonance imaging (fMRI) data analyses (Power et al., 2012), particularly when related to ASD (Tyszka et al., 2014). More specifically, there is an analytic pipeline following a “one size fits all model” under assumptions of normality, linearity and stationarity in the imaging data that does not necessarily conform to the characteristics inherent in the variability of such data. Part of the problem stems from the pervasive effects of involuntary head motion on all measures of morphometry and functions derived from structural MRI or resting state fMRI data (rs-fMRI). As such, fMRI experiments require maximal dampening of head movements that may occur during the scanning session (i.e., while lying inside the scanner) to prevent artifacts due to involuntary movements (Deen and Pelphrey, 2012; Power et al., 2012; Tyszka et al., 2014). Yet, even upon padding the head during the scan to minimize movement, these minute fluctuations in head motion are detectable and known to confound the data if no motion correction procedures are in place (Friston et al., 1996; Hutton et al., 2002; Jenkinson et al., 2002). This problem often leads to the removal of large portions of datasets so as to enable statistical inferential analyses. Furthermore, recent work underscores the importance of not making *a priori* statistical assumptions about the underlying stochastic features of biophysical rhythms harnessed from the nervous systems (Torres, 2011, 2013a; Torres et al., 2013a, 2016a). In particular, such work demonstrates that when empirically estimated, the probability distributions that characterize such signals are generally not normal; rather, they are subject to non-linear and stochastic variations inherently present in signals derived from complex systems. These biophysical signals include those derived from fMRI involuntary head-motion related data (Eklund et al., 2016; Torres and Denisova, 2016).

**Figure 1:**
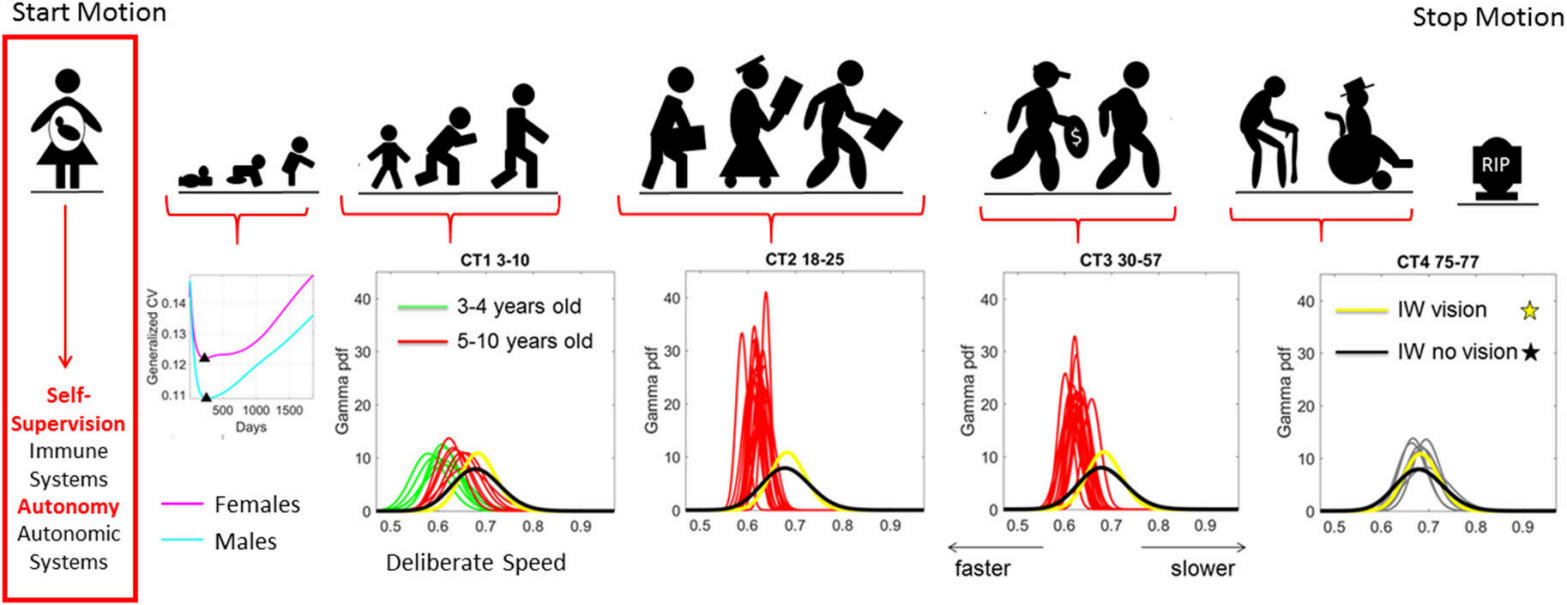
Age dependent shifts in the non-Gaussian stochastic signatures of motion variability. Physical motion starts from conception and ends with death. We propose that during early stages of life the nascent immune and autonomic systems scaffold self-supervision and autonomy, respectively thus endowing the nervous systems with features to be poised to grow and mature intelligence by partly supervising and using its own feedback to learn and adapt. As such, the variability inherently present in the biorhythmic motions of the person—e.g., in deliberate voluntary motions, spontaneous involuntary motions and the inevitable autonomic motions serve as a form of kinesthetic feedback from various systems. It is our finding that these motions are not characterized by symmetric distributions with non-stationary properties. Rather, the probability density functions empirically derived from actual physical data are skewed; shift skewness with aging and the rates of change of the shifts in skewness change within different age groups. For example, early in neonatal stages the female and male phenotypes separate according to the generalized coefficient of variation (CV) of their rates of growth, which is reflected as well in their rate of change of motion stochastic signatures (Torres et al., 2016b) [Data obtained from 26,985 babies per summary point (13,623 girls, 13,362 boys) publicly available from the methods to build the WHO-CDC Growth Charts]. Babies were longitudinally tracked for 24 months upon which cross-sectional data was used to build the charts up to 5 years of age (Kuczmarski et al., 2002; de Onis and Onyango, 2003). Inset highlights the non-Gaussian nature of the variability of this parameter of physical growth and the inflecion point attained earlier (at 224 days) in females than males (at 252 days). Later on in life such sex differences are less obvious (Torres et al., 2013b), but using the fluctuations in motion parameters (e.g., those changing in cross-sectional data spanning 3–77 years of age) can be informative of subtle differences in speed micro-movements denoting different degrees of skewness and dispersion along with different age-dependent rates of change in this stochastic signatures (data extracted from controls (CT) in 176 participants reported in Torres et al., 2016a) Yellow and black PDFs are from a deafferented participant for reference of a system without (or very poor) kinesthetic reafference manifested in the typically aging elderly.

Considering the inherent nature of the empirical data rather than *a priori* imposing theoretical assumptions for statistical inference seems particularly relevant when analyzing cross-sectional data from the population at large. Neurodevelopment is, indeed, non-uniform and highly non-linear in its early stages (Torres et al., 2016b), with the statistical properties of biorhythms from the developing nervous systems changing dramatically with age (Figure 1) (Torres et al., 2013a, 2016a). In particular, the degree to which spontaneous involuntary fluctuations in the nascent nervous systems can be dampened on command is in itself a sign of maturity (Torres et al., 2013a), as the nervous systems transition into more stable states. In the case of ASD and other neurodevelopmental disorders, the coping nature of the nervous systems adds a layer of instability that can be tracked through the assessment of involuntary motions (Torres, 2013a), particularly those that are still present in excess in the system despite instructions to remain still (Torres and Denisova, 2016). Indeed, recent results on the role of head motion micro-movements during rs-fMRI revealed elevated levels of noise-to-signal ratio (NSR) in the ASD population at large (Torres and Denisova, 2016). These elevated NSR in involuntary head micro-movements were detected with or without medication intake, suggesting that the presence of involuntary motions with excess NSR levels could serve as an important biological feature of nervous systems with developmental problems. In addition, this previous work illustrated differences between individuals as a function of medication intake (Torres and Denisova, 2016); a comparison dominated by a cohort consisting of majority males participants.

The prior work, however, did not have a sufficiently large number of female participants to examine if male participants primarily drove the elevated NSR, or if the females with a diagnosis of ASD/or AS also have inherently elevated levels of NSR. If so, this signature of stochastic motor variability may provide a route of non-invasive diagnosis, one that may tap into underlying symptomatology associated with a diagnosis of ASD in females. Within the context of resting state imaging studies involving ASD participants, questions have therefore been raised over claims on connectivity and morphometry variation as individuals with a disorder of the nervous system—including those considered a “mental illness” by the DSM (American Psychiatric Association, 2013)—often move more, which impacts statistical inferences and interpretations made (Pardoe et al., 2016).

Given the pervasive noisy and random somatic motor micro-movements signal in ASD across sex and ages (Torres et al., 2013b), severity (Torres and Denisova, 2016) and levels of motor control (voluntary, Torres et al., 2013a; automatic, Torres et al., 2016c), autonomic (Torres and Lande, 2015; Kalampratsidou and Torres, 2016; Torres et al., 2016b), the present work aimed to investigate if involuntary micro-movements of head motion recorded within the scanner had a statistically different rate of noise accumulation in ASD *females* in relation to TD control females. Further, this was examined in light of male-specific ASD-TD differential patterns to consider the impact of gender. For the purposes of our inquiry, it was not as important to consider if the person affected with ASD moved more (since we suspected they did and others corroborated that guess already in affected adults, Tyszka et al., 2014). The question is whether the continuous random process that we used to characterize those fluctuations in head motion amplitude (as spike trains) revealed higher cumulative effects of noise and randomness in females with ASD, (including as well those with a DSM-IV AS-related diagnosis) than in female controls. We report evidence that the ABIDE data sets contain information of use to help define the ASD female phenotype.

## Methods

### Demographics of ABIDE I and ABIDE II

All datasets included in this study are from the Autism Brain Imaging Data Exchange (ABIDE) databases: ABIDE I (http://fcon_1000.projects.nitrc.org/indi/abide/abide_I.html) and ABIDE II (http://fcon_1000.projects.nitrc.org/indi/abide/abide_II.html). The work is in compliance with Frontiers guideline on the use of human subject's data. To that end, quoting from ABIDE “In accordance with HIPAA guidelines and 1,000 Functional Connectomes Project/INDI protocols, all datasets have been anonymized, with no protected health information included.”

**Figure 2:**
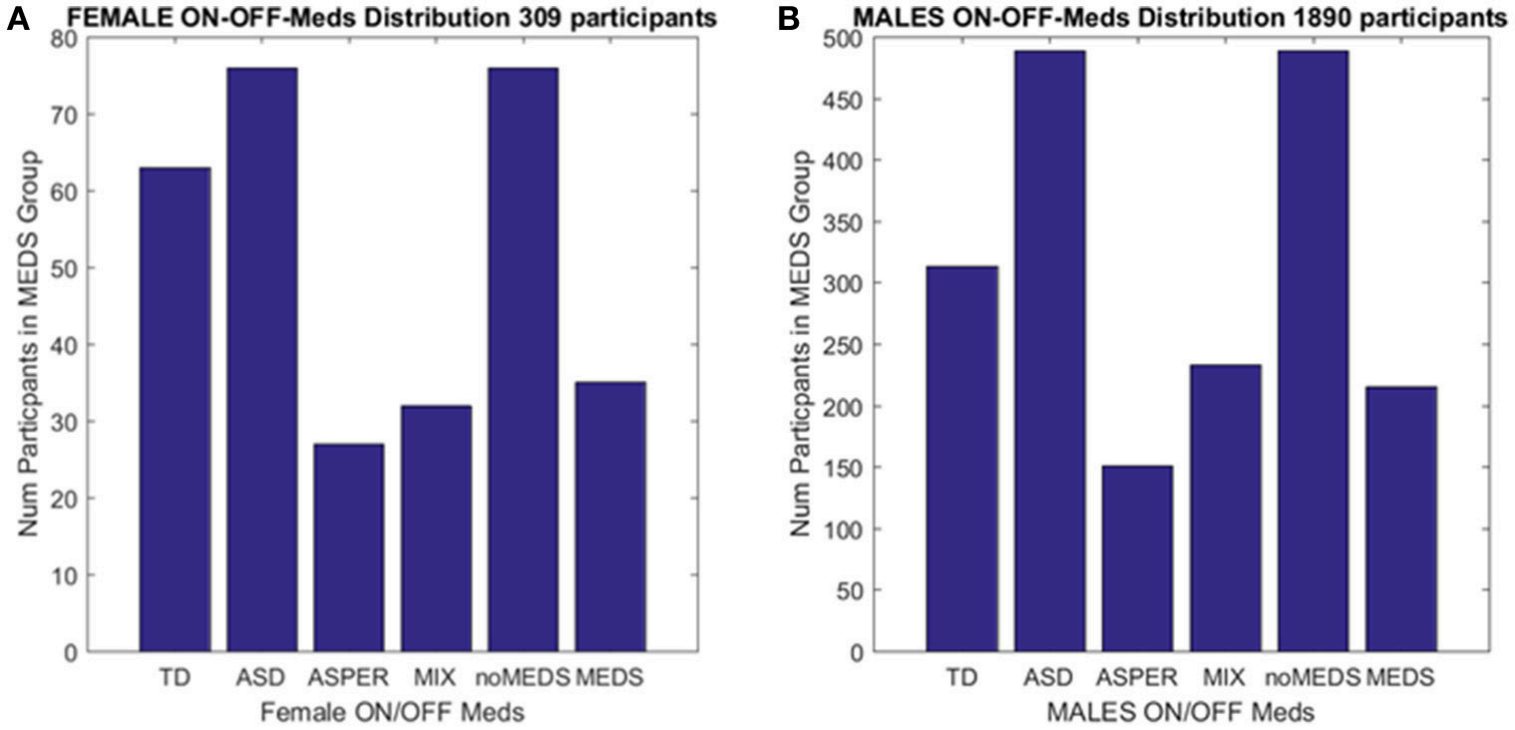
Inclusion/Exclusion criteria for the ABIDE I and II data sets used in this study. **(A)** Females: TD females are from ABIDE I and include all individuals with no medication intake only from ABIDE I sites that reported medication intake in the demographics were included as the group size covered the proper age range and comparable number of participants to those with ASD. ASD females are from column 1 DSM of demographics records across ABIDE I and II with a diagnosis of ASD; DSM-IV-TR AS diagnosis of column 2 of demographics records; MIX includes all AS, PDDNOS, PDD from column 2 DSM-IV (no ASD from DSM IV). NoMEDS refers to all with a diagnosis of ASD (column 1 and 2 of the demographics records), all with a diagnosis of AS or PDDNOS or PDD who were not on medication (i.e., from all sites that reported medications). MEDS were as before, all participants with a diagnosis but not on medication. **(B)** The same as **(A)** for males.

Collectively, these open access databases contain datasets with a much larger number of females (and males) than one could find in any given single study in the literature. The breakdown of demographics used in the present study is summarized in Figure 2. The study includes four main comparisons:

1. *ASD, AS, TD*: Comparison of stochastic signatures of head micro-movements extracted from individuals who have a formal DSM diagnosis of ASD, a DSM-IV-TR (American Psychiatric Association, 1994) diagnosis of AS and TD controls, but are not on medication.
2. *Medication vs. no Medication*: Comparison of individuals with a diagnosis who reported medication use *vs*. those who did not report medication use.
3. *ADOS-2 vs. ADOS-G*: Comparison of ADOS-2 and ADOS-G scores (Lord et al., 1989, 2000) whenever available for set in (1) above.
4. *Females vs. Males*: Comparison of females *vs*. males according to the above mentioned metrics and selected across ABIDE based on the inclusion/exclusion criterion next defined.

### Inclusion/exclusion criterion

We included those sites in ABIDE I and II that contained information regarding participant medication intake (Table 1 of the Supplementary Material lists sites with summary information). From those sites, we first isolated female individuals who did not take medication (*n* = 76). From these individuals, we isolated those with a diagnosis of ASD and those with a diagnosis of either AS (*n* = 27), or a mixed diagnosis of AS or a pervasive developmental disorder not otherwise specified PDDNOS/PDD (*n* = 32). ABIDE I was published before the DSM-5 (American Psychiatric Association, 2013) was released and only reports information as per DSM-IV-TR (American Psychiatric Association, 1994) while ABIDE II reports both DSM-IV-TR and DSM-5 diagnostic information. Due to the augmentation of terminology in DSM-5 (American Psychiatric Association, 2013) leading to putative overlap between ASD and AS, the ASD individuals were from the non-DSM-IV column of the demographics data set. The AS individuals (and diagnosis) were isolated using the next column representing DSM-IV-TR classification only. Thus, the main question was whether the two groups (non-DSM-IV ASD and the DSM-IV AS) were in any way distinguishable. Then we examined 63 age-matched (TD) females, a group of comparable size, from ABIDE I as the control group.

A second level comparison was to select all participants with a given diagnosis (i.e., ASD from the first DSM column, ASD from the second column with the DSM-IV diagnosis and those with AS, PDDNOS, PDD) who were on medication (*n* = 76) and those who were not on medication (*n* = 35). Here, the goal was to compare their involuntary head micro-movements signatures and ask if medication intake in the *females* had an effect on the stochastic signatures of the head motions. Figure 2 summarizes these demographics.

Lastly, we compared ADOS-2 and ADOS-G scores, whenever available, for the groups above and included the males selected under the same criteria for this comparison (Table 2 of the Supplementary Material lists sites with summary information). The idea was to uncover differential patterns (if any) between female-female statistically significant differences and male-male statistically significant differences. Summary of these results and the levels of statistical significance are shown in Tables 1, 2 of the Supplementary Material.

### Motion extraction

Motion extraction was performed using the Analysis of Functional NeuroImages (AFNI) software packages (Cox, 1996). Single subject processing scripts were generated using the afni_proc.py interface^1^. Skull stripping was performed on anatomical data and functional EPI data were co-registered to anatomical images. The median was used as the EPI base in alignment. Motion parameters, 3 translational (x, y, and z) and 3 rotational (pitch-about the x axis, roll-about the y axis, and yaw- about the z axis), from EPI time-series registration was saved.

### Statistical analyses

In the present work we assess the scan-by-scan velocity-dependent variations in the linear displacement and in the angular rotations of the head during rs-fMRI sessions. The analyses specifically refer to the stochastic signatures of the micro-movements (as generally defined below), their accumulation and empirically estimated statistical features under a statistical platform for individualized behavioral analysis (SPIBA). In the specific case of rs-fMRI data, the data types are not the original head motions *per se*, but rather derivative information pulled out from the original time series that the head-motion extraction methods create (Friston et al., 1995; Worsley and Friston, 1995). The commonly used methods to estimate volume-to-volume head movements from fMRI data were therefore used to obtain the original time series of (raw) head motion data (see section Methods for head motion extraction above).

### Micro-movements as a new waveform data type for analyses of motions embedded in the biorhythms harnessed from the nervous systems

Given the disparate sampling resolutions (SR) across sites reporting data to ABIDE, we here use a data type that is insensitive to the differences in stochastic processes that such different SR give rise to Caballero et al. (2017). The micro-movements (see below) are a new waveform introduced earlier to analyze motion data from various sensors used in motion caption sampling with different degrees of accuracy, frequency and temporal resolutions (Torres et al., 2013a). Instead of examining a time series of time dependent values, we rather focus on a waveform of the fluctuations in signal amplitude in the order in which the changes in the peaks of the signal occur. In the present work we use the raw linear and angular speeds extracted from the imaging data to build the micro-movements. To that end, we examine the changes in amplitude in a dynamic-independent fashion.

To derive the micro-movements, we obtained the series of local peaks (speed maxima) and divided them pointwise by the sum of the speed maximum value and the local average speed between the two minima,

(1) $$\begin{array}{r} NormSpeedMax=\frac{SpeedMax}{SpeedMax+AvrgSpeed} \end{array}$$

**Figure 3:**
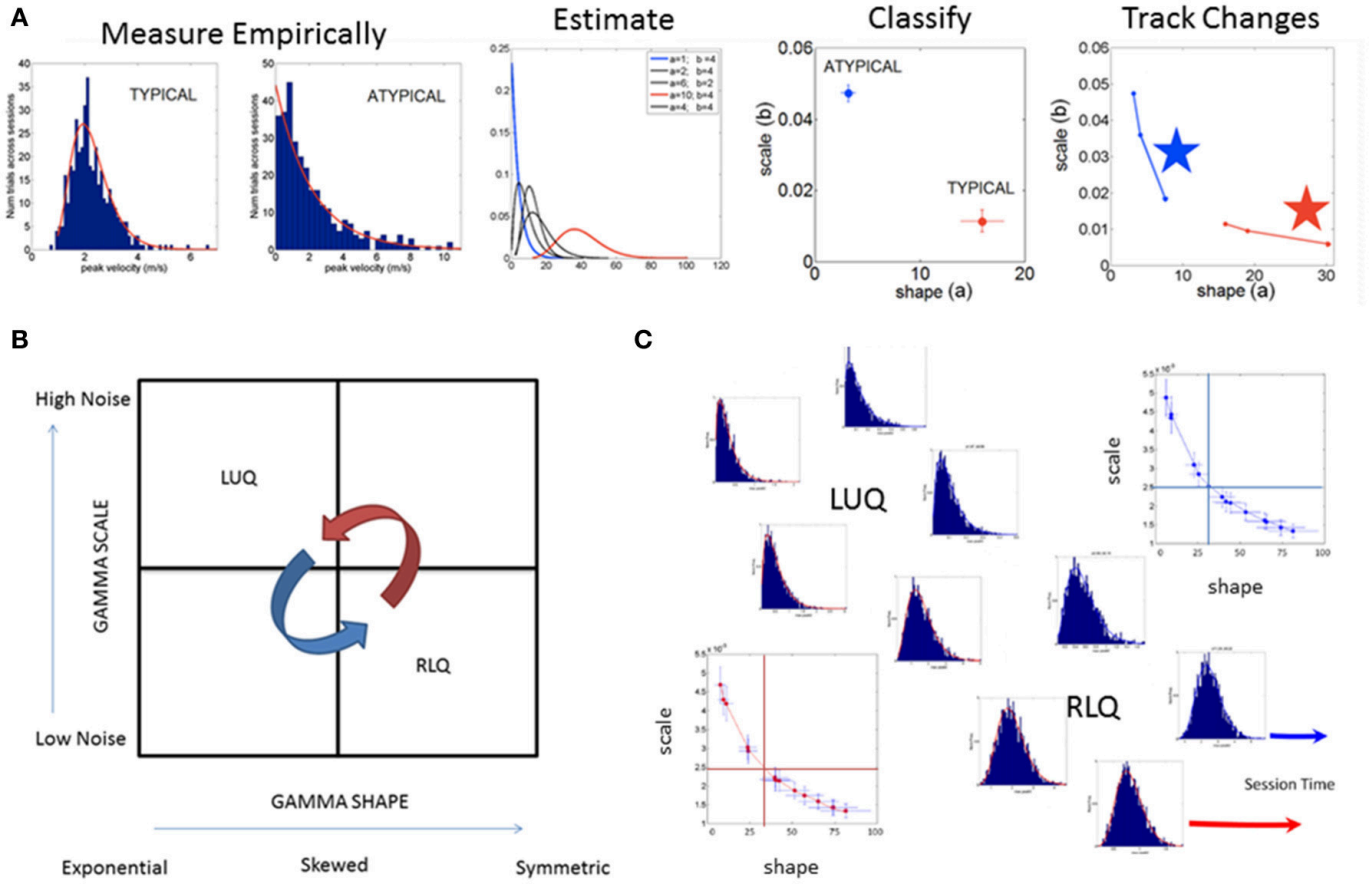
SPIBA using the Gamma process for statistical inference and interpretation of biophysical data. **(A)** Obtain frequency histograms of biophysical parameter and derive micro-movements from the waveform. Empirically estimate the PDF's using maximum likelihood estimation with high confidence and plot the estimated parameters on the Gamma plane. **(B)** The Gamma plane statistical inference for interpretation of biophysical data, (e.g., the biophysical rhythms harnessed from the Central and Peripheral Nervous Systems) is shown here in schematic form. The empirical estimation of the shape and scale Gamma parameters has provided a range of empirical data from movements encompassing a range of voluntary control levels (e.g., autonomic, spontaneous, automatic, involuntary and voluntary). Along this gradient we have profiled the autistic phenotype and found empirical evidence for the prevalence of the Exponential distribution SHAPE value of 1 to the left of the shape-axis. In contrast, the typically developed young participants tend to manifest symmetric shapes to the right of the SHAPE-axis, with skewed distributions between these two extremes prevalent across the adult population at large. Along the SCALE-axis (denoting the noise to signal ratio (NSR) of the biophysical rhythms from movements comprising multiple levels of control) the autistic population remains high in ranges of NSR in relation to the typical controls with lower levels in the steady state regimes of a task (i.e., when the person is proficient at it). This empirical evaluation of human biorhythms harnessed during natural behaviors defines two quadrants of interest to track in any experimental setting involving individualized behavioral analyses: the left upper quadrant of the Gamma plane (LUQ) and the right lower quadrant (RLQ) of the Gamma plane. Each quadrant provides (theoretical) statistical inference information amenable to interpret the actual biophysical data. The subdivision has also been used to characterize and map out the statistical ranges of human behavior with pathologies of the nervous systems in relation to normative data from typical fellows. **(C)** Different scenarios of the Gamma plane and its statistical-inference quadrants are shown in schematic form to invite its use for the tracking of stochastic trajectories of a given individual derived during a given session of a given study. The longitudinal evolutions of the probability distribution functions from the LUQ to the RLQ are important to consider in individualized neurodevelopmental data but also possible to track in scenarios comprising cross-sectional data (such as the present one).

The spike trains of amplitude fluctuations derived from this normalized version of the raw data are the waveform used as input to the SPIBA. We combine this waveform with a Gamma process to empirically estimate the Gamma parameters and track their values on the Gamma parameter plane, compute the probability distribution functions (PDFs), obtain the Gamma moments and the summary statistics (see Figure 3).

**Figure 4:**
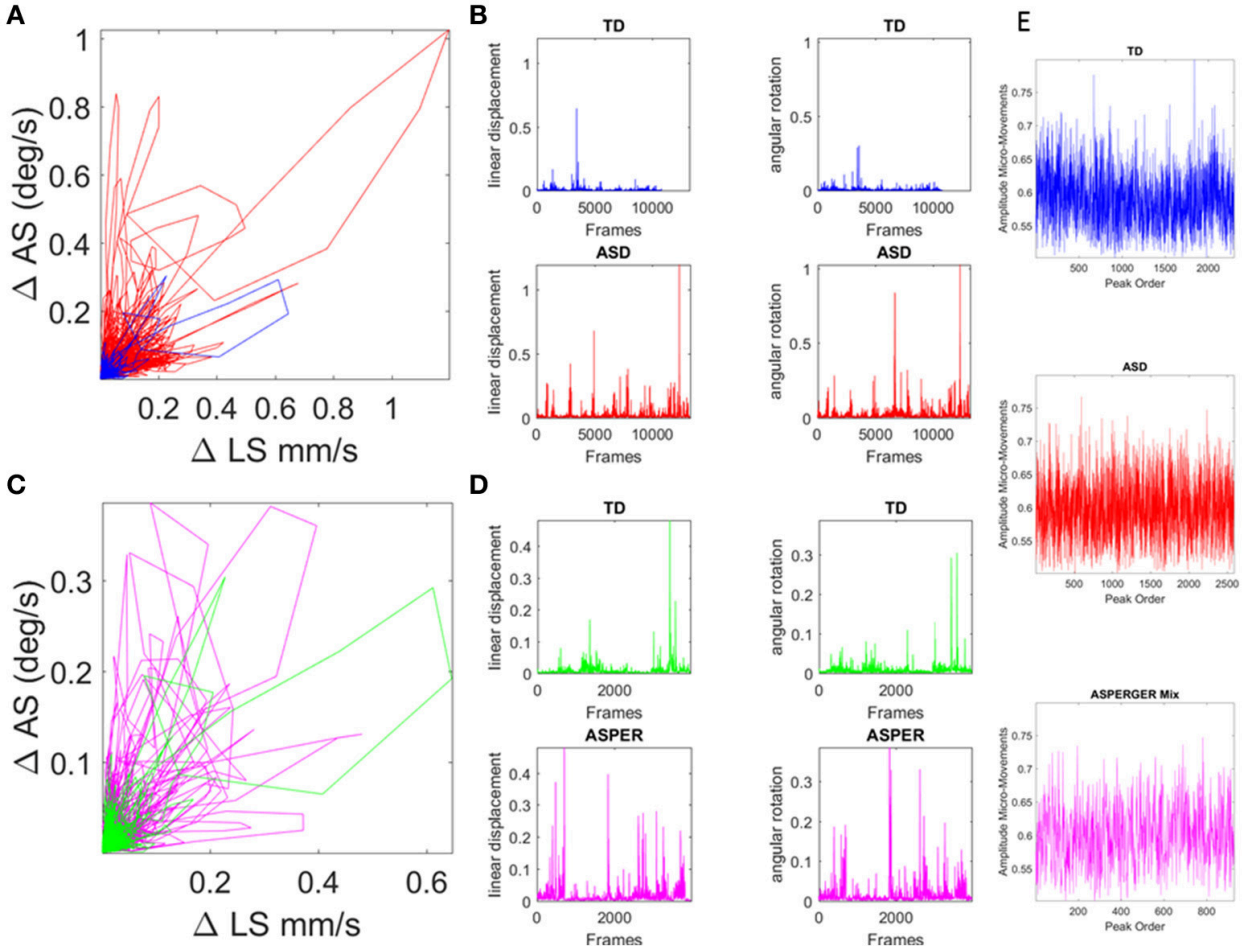
Involuntary head excursions were detected during the resting state fMRI sessions. **(A)** Linear displacements vs. angular rotations of the head contrasting ASD females vs. age-matched TD controls. **(B)** Raw data consisting of linear speed and angular speed extracted from the position and orientation tri-axial trajectories contrasting TD vs. ASD participants. **(C,D)** Same as in **(A,B)** for the females of ABIDE I and II with a DSM-IV diagnosis of AS and PDDNOS vs. the age-matched TD controls in **(A)**. **(E)** Micro-movements extracted from rs-fMRI head linear displacements in TD, ASD, and AS combined with PDDNOS participants.

Presented in prior work (Torres, 2011, 2013a; Torres et al., 2013a, 2016a,c; Torres and Denisova, 2016) and patent pending technology (Torres and Jose, 2012), the micro-movement approach examines the orderly series of peaks and valleys across biophysical data continuously registered from physiological sensors, from which such spikes can be extracted. Specifically, the fluctuations in amplitude (and timing when the instrument's sampling resolution is uniform across the data set) of such spikes are assumed to characterize a continuous random process where events in the past may (or may not) accumulate evidence toward prediction of future events (see Figure 4). Figure 4 provides a summary of the data types used in the stochastic analyses with sample raw data in Figures 4A–D, and micro-movements plots in Figure 4E.

This method has been applied to other biorhythms harnessed non-invasively from various processes of the nervous systems ranging from deliberate-voluntary to spontaneous-automatic, spontaneous-involuntary, to inevitable-autonomic (e.g., output from EEG, output from ECG, skin temperature probes, output from inertial measurement units, output from electromagnetic sensors, output from camera based systems, among others, Torres (2013b), Torres et al. (2013a), Kalampratsidou and Torres (2017), Ryu and Torres (2017), and Whyatt and Torres (2017).

Within this framework, the rate of change of raw linear displacement of the head position was obtained in vector form (a three-dimensional velocity field over time). For each velocity vector the Euclidean norm was used to obtain the magnitude of each element in this scalar field over time, i.e., the linear speed temporal profile corresponding to the given session (denoted LS). The time-series of the LS values were then plotted for each participant as a linear speed profile where each unit time depends on the scan specs (frames per second in Hz) across the lengths of the scanning sessions (plotted in Figures 4A,B for the age-matched TD vs. ASD representative samples and in Figures 4C,D for age-matched TD vs. AS and PDDNOS participants).

The fluctuations in amplitude (of LS maxima) were then normalized as in Torres (2011) and Torres et al. (2013a), using equation 1 above, scaled between 0 and 1 to account for allometric (head or body size) effects in cross-sectional data from the population at large (Lleonart et al., 2000). This standardized way of examining physiological signals (the micro-movements data type) further permits grouping of the movement data using clinical and demographic features of participants with heterogeneous demographics and phenotypic information (Torres and Jose, 2012).

The normalized peaks in the order in which they appeared are plotted in Figures 4E and for each type of participant. This waveform then served as input to a Gamma process and stochastic characterizations of their fluctuations in amplitude were used to provide a signature of the ASD, AS, and TD groups. Thus, we examined the continuous spike train data of orderly speed amplitude shifts as a Gamma process under the general rubric of a Poisson Random Process (PRP), assuming independent and identically distributed (IID) random variables. This assumption will be relaxed in future work; but for the purposes of our examination concerning the traditional *a priori* assumption of normality in such biophysical data, it should suffice to consider the simpler case of a point process where the distributions have various degrees of dispersion, skewness, i.e., are not normal and different kurtosis.

Briefly, the Gamma probability distribution function is given by: $y=f\left(x|a,b\right)=\frac{1}{\Gamma \left(a\right)b^{a}}x^{a-1}e^{\frac{-x}{b}}$, in which *a* is the shape parameter, *b* is the scale parameter, and Γ is the Gamma function (Ross, 1996). We used in-house developed software and MATLAB version 8.3 (R2014a) (The MathWorks, Inc., Natick, MA) functions to estimate the Gamma parameters and corresponding PDF (and CDF) using maximum likelihood estimation (MLE) with 95% Confidence Intervals (CIs). To that end, we compared different families of probability distributions (e.g., the Gaussian, Normal, Lognormal, Exponential and Gamma) and chose the best fit in an MLE sense. Owing to our prior work using the ABIDE sets (Torres and Denisova, 2016) we were able to determine that the Gamma had the best fit in an MLE sense. Of particular importance, the (NSR), a.k.a. the Fano Factor (FF, Fano, 1947) is obtained from the empirically estimated Gamma variance divided by the empirically estimated Gamma mean. The Gamma mean is given by μ = *a* · *b* and the Gamma variance is given by σ^2^ = *a* · *b*^2^. The NSR in this case is also the Gamma scale parameter since σ2μ=a·b2a·b=b (Ross, 1996). This is important as we will be assessing the levels of noise in relation to the empirical estimation of the Gamma parameters from the data as a function of group type. Higher levels of noise in the left upper quadrant of the Gamma plane (Gamma-LUQ) will correspond to increases of the *b scale* parameter along the vertical axes of the Gamma plane; whereas lower levels of noise in the right lower quadrant (Gamma-RLQ) will correspond to lower values along the *scale* axis of the Gamma plane. This is shown in Figure 3B in schematic form with schematic examples of stochastic trajectory evolution across the quadrants of interest in Figure 3C. The quadrant's limiting values (represented by the quadrant-dividing lines) are derived from the stochastic signatures of the evolution set as the median values of the scale or shape empirically estimated parameters.

It is also important to emphasize that when the *shape* parameter *a* of the Gamma family *a* = 1 at the Gamma-LUQ, the data follows the memory-less Exponential probability distribution. This is the most random distribution whereby events in the past do not accumulate information predictive of events in the future (Ross, 1996). Larger values of the shape parameter toward the Gamma-RLQ tend toward the symmetric distributions, with a variety of skewed distributions between the two extremes.

The scatter of points on the log-log Gamma plane gives rise to a power-law relation between the shape and the dispersion of the distributions [the scale parameter or Noise-to-Signal Ratio (NSR)]. The extent to which the scatter points deviate from this pattern can be quantified. To that end, it is possible to measure the residuals from the linear polynomial fit (denoted here as delta) and obtain a parameter plane involving the delta values vs. the corresponding NSR for each point (representing a participant) in the scatter. This information can thus give rise to statistically driven clusters (Nguyen et al., 2016) to classify various subtypes of patients.

Here we adopt such a metric (that we introduced in Nguyen et al., 2016 and adapted to rs-fMRI data from ABIDE in Torres and Denisova, 2016) to ask if the females of the ABIDE sites that reported medication intake follow any type of automatic sub-group classification. Note here that we do not include females for whom medication status was unknown. To that end, we integrated information from the NSR and the delta residuals from the linear polynomial fit (power-law relation) associated to the scatter of the log-shape and log-scale values on the Gamma plane and will examine the ranges of parameter values within each group. In the text we will refer to the level of randomness in the empirically estimated shape parameter (when close to *a* = 1), the limiting Exponential case; or we will point out increasing values of the shape parameter toward more symmetric distributions tending to the Gaussian limiting case. Likewise we will refer to higher or lower NSR levels according to the empirically estimated *b* Gamma scale parameter value relative to the age-matched TD control values (as normative data) Figure 3A.

## Results

### Significant differences in physical head excursions distinguish ASD and AS females from TD females

We examined the relative head excursions during rs-fMRI sessions for each individual female in the cohort. To that end, the cumulative sum of speed values over all frames was obtained (i.e., the physical path length the head traveled) and divided by the number of frames for each participant. The rate of change raw data (before normalization) can be seen for TD vs. ASD in Figure 4A with their speed profiles in Figure 4B. Figure 4C shows the comparison for the TD age-matched vs. the AS. Figure 4D shows the corresponding speed profiles for each group.

**Figure 5:**
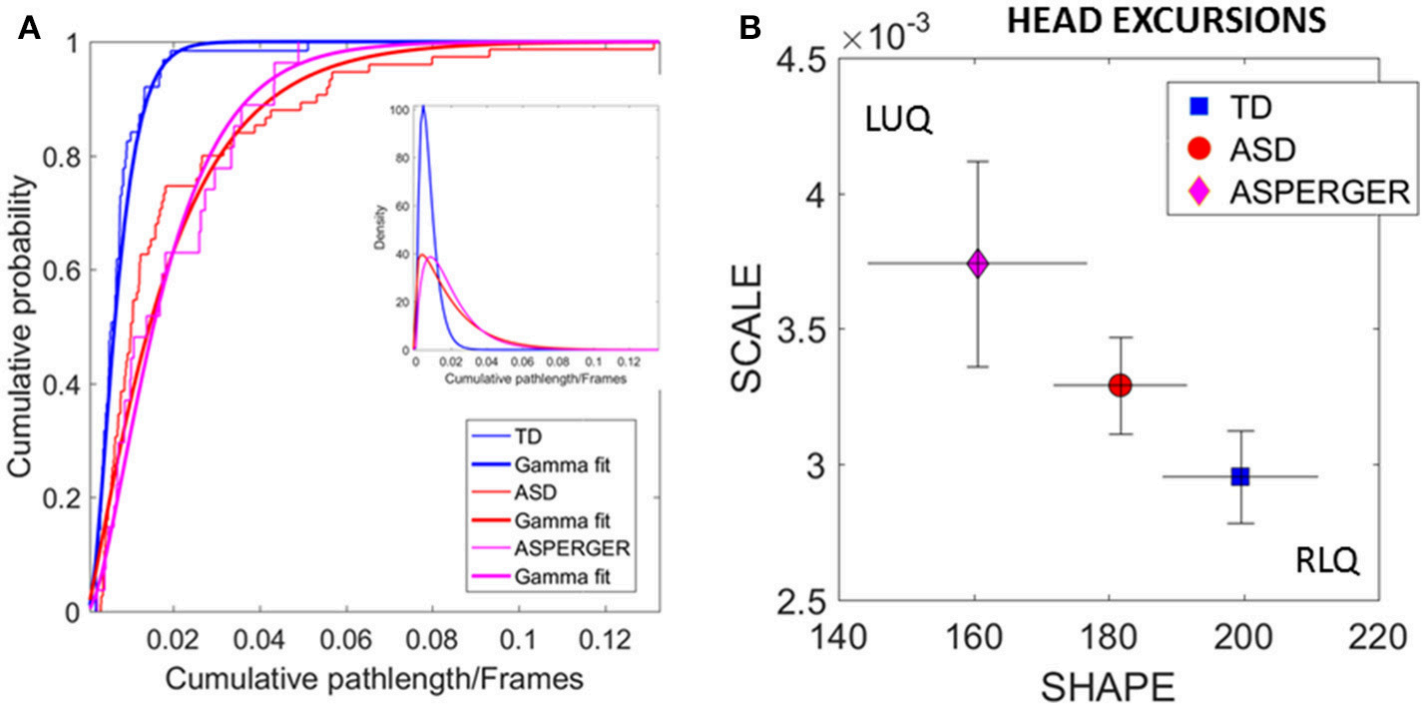
Differences in relative physical head excursions manifest in female participants during rs-fMRI session upon instruction to remain still. **(A)** The empirical cumulative distribution functions (eCDFs) estimated using Gamma fits to the empirical data pooled across all subjects per pre-labeled group separate age-matched TD participants from ASD and AS. Note that eCDFs from ASD and AS participants also separate, but the separation has non-statistical significance (see text). The inset shows the estimated Gamma PDFs. **(B)** Using the SPIBA Gamma process the involuntary head micro-movements of each labeled cohort localize AS and ASD on the LUQ of the Gamma plane with more elevated NSR relative to TD controls localized on the RLQ of the Gamma plane.

The distributions of the relative head excursion ratios were well fit by the continuous family of Gamma PDFs. Figure 5A shows the empirical cumulative probability distribution (eCDFs) and the estimated CDFs for all three groups of age-matched females. The inset shows the estimated first and second Gamma PDFs. Figure 5B shows the signatures localized on the Gamma parameter plane. The estimated Gamma moments were also obtained and the results are summarized in Table 3 of the Supplementary Material.

We next focus on the linear displacements. We compare the *relative* head excursions pairwise across each female group. To that end we used the non-parametric Mann-Whitney-Wilcoxon U rank-sum test. We found statistically significant differences between the pooled data of ASD females and age-matched TD female controls (rank sum test *p* < 2.22e-06), with notably more head movement (as recorded by physical head excursion) for the ASD female group (see Figures 4C,D vs. Figures 4A,B). This was further identified in a significant comparison between the AS females, and age-matched TD female controls (rank sum test *p* < 1.95e-05), but no significant differences were found in the length of head excursions between ASD and AS females (*p* < 0.39). Furthermore, we used the Kolmogorov-Smirnov goodness-of-fit hypothesis test from MATLAB to compare two empirically estimated eCDFs. The pairwise comparison for the relative head excursion parameter yielded significant differences for TD vs. ASD (*p* < 3.42e-05) and for TD vs. AS (*p* < 1.72e-04) but was not significant for ASD vs. AS (*p* < 0.54). The ASD vs. AS proximity in the distributions can be appreciated in Figure 5A and the overlapping confidence intervals in Figure 5B, along with their separation form TD controls.

Similar analyses were performed to compare all females with a diagnosis on medication vs. those who reported not being on medications. No significant differences were found between the ASD and AS groups of females (*p* < 0.31).

### Data-driven separation of ABIDE females

**Figure 6:**
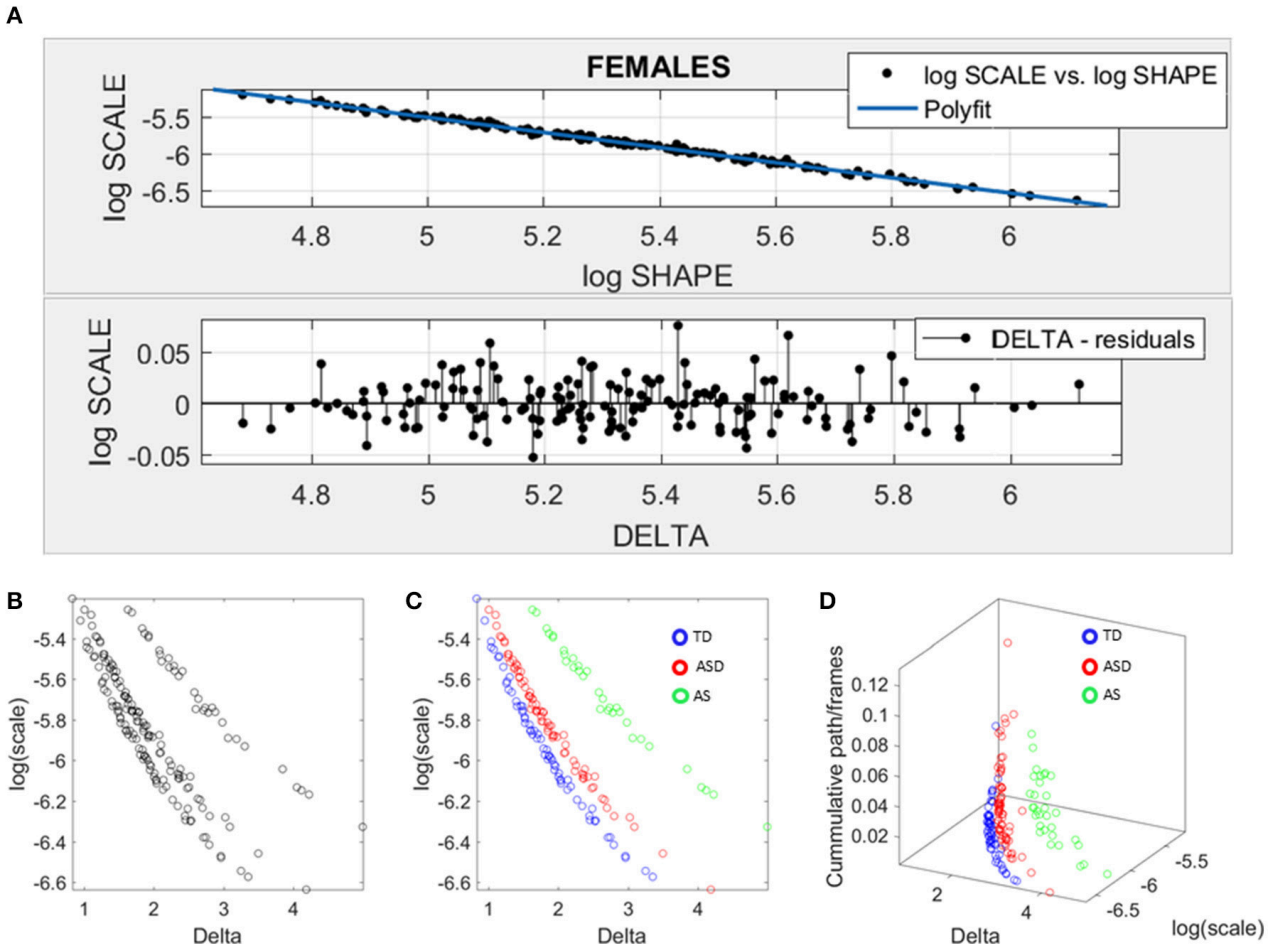
Data-driven approach for cluster detection based on stochastic properties of the head micro-movements data of the females in ABIDE. **(A)** Individually estimated Gamma probability distributions of the females and power relation fit using polynomial of degree 1 on the log-shape vs. log-scale parameter plane (top panel). Bottom panel shows the residuals (delta) obtained from the error between the polynomial fit and the actual scatter points. **(B)** Parameter plane distinguishes three clusters along the Delta vs. log (scale) or noise to signal ratio (NSR). **(C)** Scatter colored by DSM labels reveal clusters congruent with the diagnosis. **(D)** Further separation of the groups emerges when using the relative head excursion (cumulative path length per frames), with the AS group singled out as the farthest apart from the age-matched TD controls.

We used SPIBA to examine the micro-movements of the head linear displacements extracted from the rs-fMRI. As explained in the methods section, these spike trains were used as inputs to a Gamma process and the Gamma shape and scale (the NSR) parameters are plotted on the Gamma plane (Figure 6A). The log-log plot of this scatter yielded a power relation, whereby a polynomial of degree 1 was fit using polyfit via the MATLAB curve fitting toolbox [Linear Model Poly1 *f*(*x*) = *p*_1_ · *x* + *p*_2_ with *p*_1_ = −1.03(−1.09, 1.02) and *p*2 = −0.369(−0.4185, −0.3194)]. The goodness of fit was SSE 0.06, Adjusted R-square 0.9962 and RMSE 0.01.

The residuals (delta) from the linear fit against the actual scatter of points were examined and plotted on the bottom panel of Figure 6A. The deltas vs. the log of the Gamma scale (NSR) were plotted on a parameter plane in the random order in which they were examined. Three groups emerged with clear separation—see Figure 6B. The scatter was subsequently colored coded according to the diagnosis label (Figure 6C). As is evidenced in Figure 6C, the ASD females separated from the AS females, while both groups separated from the TD controls. We underscore here that the bottom panel of Figure 6A contains the deltas in random order. There is no a priori-selection that leads to Figure 6B systematic separation. It is rather a systematic separation that *self emerges* without the use of the labels (*unsupervised mode*). Then Figure 6C is colored with the labels (*supervised mode*). Further, the cumulative path/frame (head excursion ratio) was plotted along the *z-axis* (Figure 6D) and the groups further separated (surprisingly) showing the AS group as the farthest apart from the age-matched TD controls.

### Data-driven separation of ABIDE males

**Figure 7:**
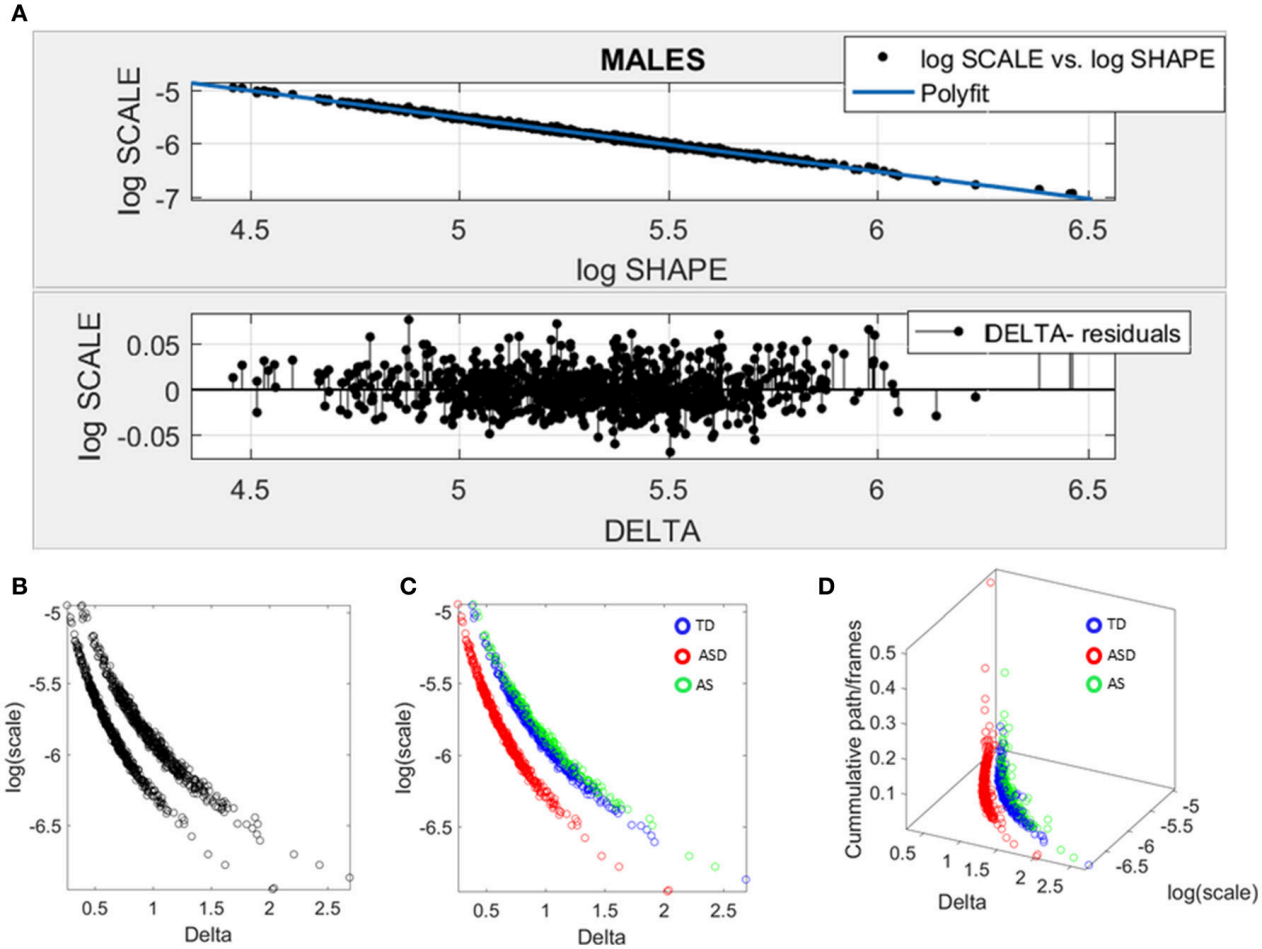
Data-driven approach for cluster detection based on stochastic properties of the head micro-movements data of the males in ABIDE. **(A)** Individually estimated Gamma probability distributions of the females and power relation fit using polynomial of degree 1 on the log-shape vs. log-scale parameter plane (top panel). Bottom panel shows the residuals (delta) obtained from the error between the polynomial fit and the actual scatter points. **(B)** Parameter plane distinguishes three clusters along the Delta vs. log (scale) or noise to signal ratio (NSR). **(C)** Scatter colored by DSM labels reveal clusters congruent with the diagnosis. **(D)** Further separation of the groups emerges when using the relative head excursion (cumulative path length per frames), with the ASD group singled out as the farthest apart from the age-matched TD controls and AS subgroup overlapping with the TD controls.

Given the results in the female cohort, the SPIBA approach paired with the Gamma process was used to examine the males across ABIDE using the same inclusion-exclusion selection criteria as with the ABIDE females. Figure 7 shows the resulting plots from these analyses. As in Figure 6A involving the females, we found that the log-log plot of this scatter yielded a power relation whereby a polynomial of degree 1 was fit using polyfit via the MATLAB curve fitting toolbox [Linear Model Poly1 *f*(*x*) = *p*_1_ · *x* + *p*_2_ with *p*_1_ = −1.02(−1.024, 1.015) and *p*2 = −0.423(−0.4364, −0.3879)]. The goodness of fit was SSE 0.488, Adjusted R-square 0.9947 and RMSE 0.02, Figure 7A.

The delta residuals in random order are plotted in Figure 7A-bottom panel. They give rise to two main groups in the *unsupervised* case plotted in Figure 7B. The supervised case in Figure 7C reveals that in the males of ABIDE, the AS group overlaps with the TD controls. It is instead the male-ASD group that falls farther apart from the controls and AS groups. This comparison revealed a marked contrast with the ABIDE females in Figure 6, suggesting that the male ASD and the female ASD are two distinct *somatic-motor* phenotypic groups.

### Impact of clinical severity

**Figure 8:**
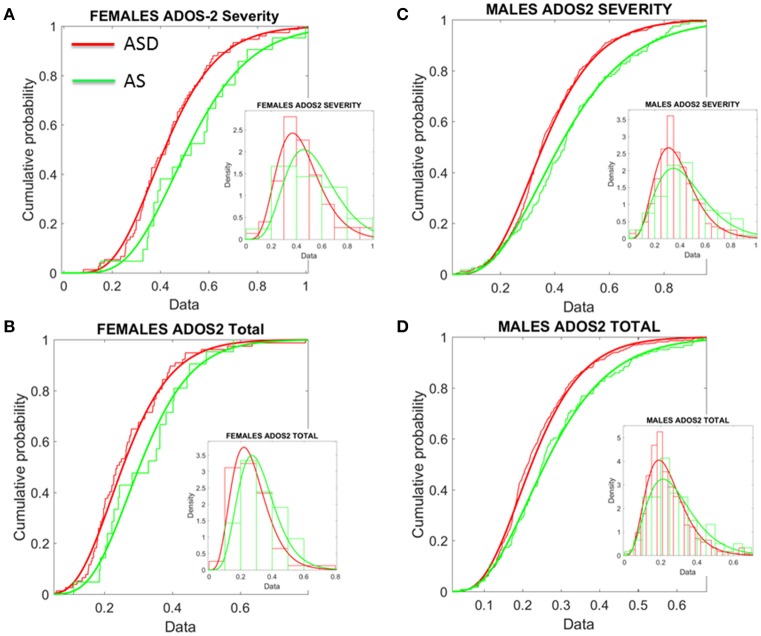
Age corrected (incremental) ADOS-2 scores mark statistically significant differences between ASD and AS observational phenotypes in the cross-sectional data form ABIDE I and II of sites that reported medication status. **(A)** Females with an AS diagnosis have higher age-corrected ADOS-2 severity scores than ASD females (Table 1 in Supplementary Material) reports the Gamma fit first (mean) and second (sigma) moments from highly skewed distribution of incremental scores considering physical age of the person at the time of the test (i.e., this is different corrective criterion than adjusting for mental age, already factored into the module selection process). **(B)** Same trend as in **(A)** for the age-corrected ADOS2-total reveals worse scores for AS females. **(C,D)** The analyses of **(A,B)** for females were performed on the males. Similar statistical features were detected for the incremental age-corrected ADOS-2 scores: skewed distributions with higher mean values for AS in relation to ASD.

Given this result, severity metrics were examined to consider the symptomatology composition of the ASD and AS cohorts. As such, ADOS-2 (Autism Diagnostic Observation Schedule, Edition 2; Lord et al., 2012) and ADOS-G (Autism Diagnostic Observation Schedule Generic; Lord et al., 2000), scores were extracted where possible, to characterize associated severity. Table 2 of the Supplementary Material lists the ABIDE sites providing such information. Operationalizing clinical diagnostic criteria stipulated via the DSM (American Psychiatric Association, 1994, 2013), these “gold standard” (Lord et al., 1989, 2000, 2012; Gotham et al., 2008), clinical tools provide standardized scoring metrics to quantify and characterize axes of ASD, whereby “higher” scores are reflective of more pronounced symptoms, thus severity. The aims were therefore (1) to examine if female ASD and AS participants with DSM-based labels could be further refined by ADOS-based severity criteria; (2) to examine if male participants with ASD and AS DSM-based labels could be further refined by ADOS-based severity criteria and (3) if ADOS-based severity in males vs. females provided further information to further integrate both clinical and research criteria with the objectively determined subtypes (see Figure 8 and Supplementary Materials for information on other ADOS-sub-scores).

To that end, the ADOS-G and ADOS-2 scores were first normalized relative to the maximum values allowed for each sub-score scale. Further, as age-related coping mechanisms in ASD appear to impact the stochastic signatures of micro-movements (Torres, 2013a; Torres et al., 2013a), the age of the participant at the time of the scan was used to correct for possible age differences due to the developing mechanisms. As such, we normalized the scores by age and set them on a 0–1 scale. These normalized scores reflect a measure obtained relative to the individual. However, due to the clinical characteristics of the ADOS-2 and ADOS-G scales (Lord et al., 2000), with no normalized population data for comparisons, it is difficult to anchor the ADOS-based scores to normative data to help interpret performance in relation to the neuro-typical population (unlike the analyses in Figures 6, 7 providing a relative metric, in relation to TD controls).

Comparisons were then made between the ADOS-2—total, severity and sub-scales (RRB–restricted repetitive behaviors and SA–social affect). As illustrated in Tables 1, 2 of the Supplementary Material, the overarching severity score and total scores were significantly different across the cohort. This pattern was further reflected in a significant difference between overarching ADOS-G total scores for each group. Yet, upon closer inspection of the metrics derived, these results illuminate a number of interesting, and somewhat puzzling findings.

First, the summative statistics, empirically derived through distribution fitting (rather than theoretically assuming normality), yielded higher average measures for each of the female and male AS sub-groups in relation to the corresponding ASD group. As measured by the ADOS-2, denoting the feature quantified by each element (sub-score and total metrics), the average was worse in AS females than in ASD females of comparable neuro-developmental age (as measured by the age corrected scale). We underscore however, that this somewhat counterintuitive finding is underpinned by empirically derived estimates of the mean and variance, rather than *a priori* assumptions of normality across the data. In particular, the parameters of interest were extracted, normalized and the probability distribution function that best characterized the distribution of the data harnessed to extract both the mean and variance (see Figure 8). These results map onto the patterning derived through empirical, objective (*unsupervised*) examination of the underlying head micro-movement during rs-fMRI (see Figure 6), whereby female AS participants are found to be notably separated from the female TD group. Indeed, the female ASD group appears to display more commonality to the TD group at this objective level.

Second, this pattern is further mirrored in “higher” ADOS-G results for the female cohort, again implying pronounced symptomatology for the female AS group in comparison to the female ASD group. Yet, despite this pervasive finding across the female cohort, mapping well onto the pattern of grouping according to stochastic signature of physiological variability, the male cohort fail to display this feature consistently across the ADOS-G parameters. In particular, at this level, the male group inverts, whereby male participants diagnosed with ASD display “higher” ADOS-G scores in relation to the corresponding AS male group—a finding that is consistent across this clinical tool i.e., sub-scales and total metrics. More in keeping with traditional expectations (i.e., more pronounced symptomatology associated with ASD), this finding may also point to the similarities we unveiled in Figure 7 between TD and AS male participants in relation to objective (*unsupervised*) profiling of the stochastic signature of micro-movements.

When examining the profile of significant differences between individuals with ASD and AS across the ADOS-2 and ADOS-G for both the female and male cohorts, further differences are highlighted. Specifically, fewer axes of both the ADOS-2 and ADOS-G significantly differentiate between female ASD and AS, whereas more consistently significant differences are found between the male ASD and AS groups (see Tables 1, 2 of the Supplementary Material). This pattern may be indicative of the sensitivity (or lack thereof) of the clinical assessment tools to quantify and classify symptomatology of ASD in females.

**Figure 9:**
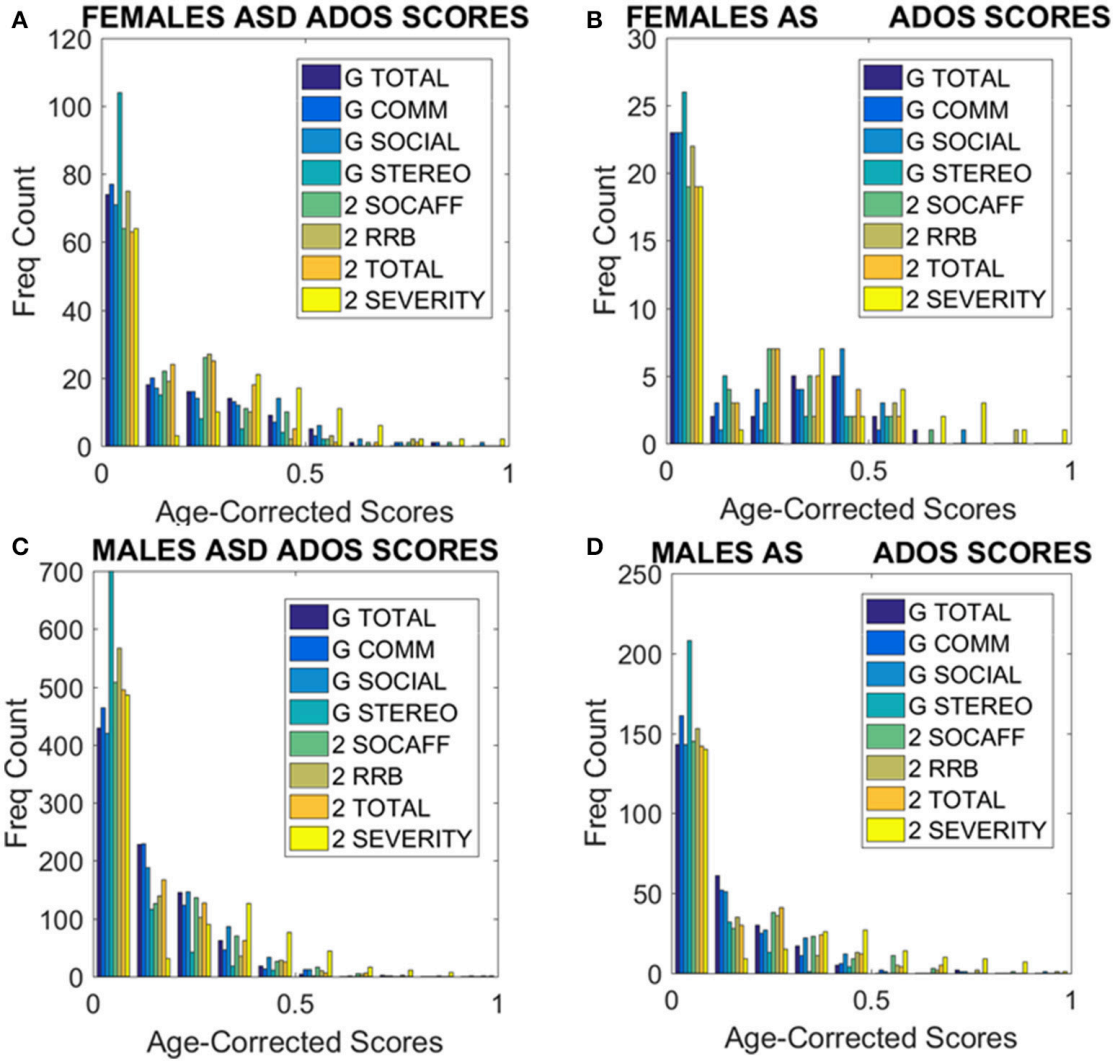
ADOS-2 and ADOS-G scores at a glance taken from all subjects we included in the analyses. **(A)** Females with a non-DSM-IV diagnosis of ASD. **(B)** Females with a DSM-IV diagnosis of AS. **(C)** ASD Males as in **(A)**. **(D)** AS Males as in **(B)**. Note the skewness of these distributions and the differences in their tails separating the two sexes. As detected by the non-parametric Rank-Sum test, the ADOS-2 severity and the ADOS-2 total do not tapper off as the individual ages, physically grows and develops neutrally at irregular rates.

Further comparison between AS males and females, and ASD males and females were performed in relation to ADOS-G and ADOS-2 scores. These are provided on Tables 4, 5 of the Supplementary Material. All ADOS-G scores yielded significant differences with higher average scores for females (suggesting higher severity). Several ADOS-2 scores also yielded statistically significant differences and higher scores on average for the females. Yet, despite empirically derived, these summary statistics are based upon different probability distribution functions (in some instances) for each sex, as shown by Supplementary Tables and Figures. Indeed, overall, the distribution of ADOS-2 and ADOS-G sub-scores in the cases of ASD and AS females have very different tails than that of the males (Figure 9). This hints at a different statistical landscape altogether for the female case. Combined, such results caution that it may be inappropriate to continue the use of a *social-behavior male ruler* as imposed by clinical tools to measure the female ASD phenotype—a feature already unveiled by the somatic-motor metrics of involuntary motion in Figures 6, 7 underlying any behavior (social or otherwise.) See additional figures in Supplementary Material which provide sub-score distributions and Tables 4 and 5 lists the outcome from the male-female comparison with the caveat (as with Tables 1, 2 above) that we do not have any reference to normative population data (i.e., preventing us from using relative population scores) to anchor these results to (i.e., while using absolute population scores).

## Discussion

Arguably, the most striking result in the present work stems from the data-driven approach that revealed automatic clustering of subgroups with fundamentally different patterns between females and males. Specifically, the head motion patterns obtained from imaging data during resting state fMRI experiments—which are commonly used to remove motion artifacts from the images—can be harnessed to serve other purposes, namely to facilitate diagnosis and classification of separable subtypes. Indeed, groups appeared on a parameter plane according to the NSR within the head-motion signal, and the extent to which the participant's stochastic signature departs from a power relation between the shape and dispersion of the empirically estimated distributions derived from their involuntary head motions. Further, groups separated according to the relative head excursions that the individual experienced while resting in the fMRI session under the instruction to remain still. In the female cohort this result pointed at the AS group as the one having the most dissimilar involuntary micro-movements' signatures from the age-matched TD controls. In contrast, the male AS group overlapped with the TD male participants, potentially more in-line with expectations. Indeed, in this instance it was the ASD subgroup that emerged as the most dissimilar with respect to the TD controls.

Such results suggest that the stochastic signature of physiological variability may provide a physical, non-invasive method to objectively characterize the ASD phenotype. In particular, this method may provide a novel insight into the functioning and expression of ASD across the female population—a cohort known to be difficult to diagnose and examine. Indeed, current discussion points toward an under-diagnosis of ASD in the female population, with a number of females potentially missing diagnosis or being misdiagnosed in the clinical field (Gould and Ashton-Smith, 2011; Wing et al., 2011), developing coping strategies or mechanisms that result in the failure to diagnose and thus provide services to these females when exclusively basing their diagnosis on a male model of social behavior.

The extent to which these somatic-motor disturbances may be captured by observational tools may be reflected in the age-corrected ADOS-2 severity and total scores that reached statistical significance for comparisons between ASD vs. AS females. Specifically, the pattern of separation between the ASD and AS groups of females (at this observational level) is reflected in a distinct *unsupervised* separation of groups in relation to underlying stochastic signature of the physiological signature. Combined, these results suggest that an increase in somatic-motor noise in AS females distinguishes this group from the ASD group (and TD group)—a distinction reflected in the clinical tool assessment. Yet, interestingly, this separation is in a—perhaps counterintuitive—direction, with more pronounced difficulties or symptomatology recorded in female AS participants. When these analyses were extended to the males under similar criteria, the separation between ASD and AS males remained strong and the age-corrected ADOS-2 severity score also separated them with statistical significance. Yet, unlike in females, the head micro-movement analyses in males did not reveal fundamental statistical differences between the TD male controls and the males with an AS DSM-IV diagnosis.

The age-corrected ADOS-G scores provided a somewhat different landscape from those of the ADOS-2. Specifically, the pattern illustrated across the female cohort was inverted for the males. As demonstrated, AS females displayed systematically *higher age-corrected ADOS-G* scores than ASD females, a trend that persisted across both the ADOS-2 *and* ADOS-G. According to clinical interpretation, such results infer worse social-related symptoms in AS females than ASD females (communication, social and stereotypic behaviors)—a pattern also reflected at the physiological level. Yet, in comparison, the male AS cohort demonstrate systematically *lower age-corrected ADOS-G* sub-scores across all categories listed in the ADOS-G (see Table 2 of the Supplementary Material); a result that is an inversion to the pattern across the female group, and indeed, an inversion to the result displayed by the male AS group examined using the ADOS-2 (See summary Figure 9 to see the results at a glance). This (implicitly) may imply that their social behavior as measured by these tests and the scores they provide (as properly corrected here by physical age) point at AS males being closer to TD controls than the ASD males. We underscore here the word “may implicitly imply” because the paper describing the ADOS-G explicitly states the need to test this inventory with typical control participants. As such, we deduce that the scores of the ADOS-G as those of the ADOS-2 are absolute, rather than derived relative to normative data.

**Figure 10:**
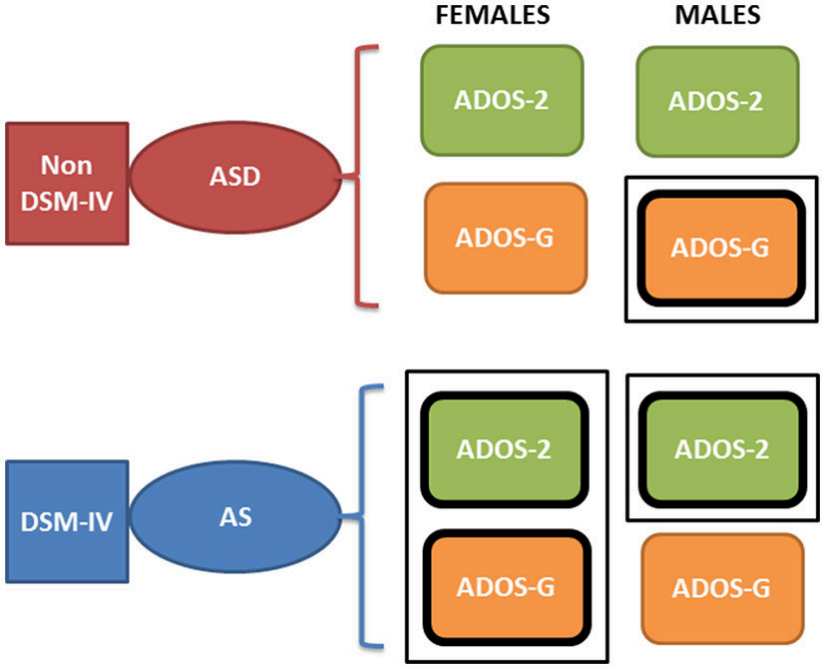
Contradictory results between the ADOS-2 and ADOS-G reported demographics in ABIDE I and II in relation to the non-DSM-IV ASD and the DSM-IV AS phenotypes. According to the theoretical population assumptions underlying the ADOS-based scoring systems, if we were to have a random draw of one male individual with a diagnosis of ASD and one male individual with an AS diagnosis from the ABIDE population that reports medication, we would find that the ASD individual is likely to be worse off than the AS under ASD-G criteria (enclosed rectangle) but better off than the AS under the ADOS-2 criteria (enclosed rectangle). In contrast, if we were to do this with females, both ADOS-based criteria would yield a better outcome score for the ASD than the AS DSM-driven phenotype (enclosed rectangle comprising both ASD-2 and ASD-G). Note here that these are the outcomes of statistical tests. We are not interpreting here the data beyond that outcome from the ABIDE data (see *p*-values and empirically estimated summary statistics in Tables 1, 2 of the Supplementary Material).

Yet, why the different pattern of results between sexes, and what can this tell us in light of the physiological metrics? First, the pattern of pronounced difficulty across the female AS cohort in relation to the ASD group may infer more pronounced symptoms in the female AS participant pool. Indeed, social-behaviors, such as those examined and quantified by the ADOS-2 and ADOS-G, intrinsically depend upon a level of motor control. As such, the result that individuals with higher levels of sensory-motor noise display “higher” scores capturing more pronounced ASD symptomatology may not be surprising. Further, it must be noted that the pattern of significant differences, across the clinical assessment are constrained to the total and severity metrics for the female cohort—perhaps reflective of the complexities associated with profiling the subtleties of ASD behaviors in the female population. Indeed, in line with physiological assessment, the one sub-scale (across both the ADOS-2 and ADOS-G) in which significant difference between the female sub-groups emerge is that of stereotyped behaviors in ADOS-G. It may be the case that this form of movement variability—both at an observational and micro-level—is a characteristic feature associated with AS in the female cohort. Secondly, the inversion of the male cohort at the level of ADOS-2 and ADOS-G is puzzling. With the ADOS-G outcomes sitting in line with the physiological metrics (i.e., with male AS participants being more in line with TD participants than those with ASD–see Figure 8), the objective physiologically driven results may place more weight on the outcomes of the ADOS-G. However, the ADOS-G criteria is (according to their authors) incomplete to completely render a diagnosis of ASD as it lacks the repetitive behavior sub-scores (Lord et al., 2000)–which we see here as the one sub-score with somewhat explicit motor component form *overt* observable behaviors that we could more directly relate to the data-driven results. On the other hand, the ADOS-2, which contains the sub-score from repetitive behaviors the ADOS-G lacks, does not align with the data-driven results based on involuntary motor issues. In fact, the males, which dominate ASD research due to the 5:1 male to female ratio, are according to the ADOS-2, better off in the non-DSM-IV ASD classification than in the DSM-IV AS classification (Figure 10, Tables 1–5 of the Supplementary Material). Yet, according to the ADOS-G, it is the opposite: the ASD males are worse off than the males with AS in all social and communication aspects. *Which one is it?*

A further element of potential concern with such observational clinical assessment tools, such as the ADOS-2 or ADOS-G aimed to operationalize the working DSM model, is the underlying assumption of a theoretical normal distribution across the population. This assumption underpins the ability for such tests to derive and report a (assumed) mean and standard deviation from their empirical computations. Yet, here the distribution of observational outcomes (i.e., those with the ADOS-2 and ADOS-G) were collated, the probability distribution that best characterized that metric *empirically estimated* (see Supplementary Material) were not symmetric. Such empirical work illustrates the inherent variability, even at this level, of ASD characteristics, with the underlying distribution across the population of scores extracted from the ABIDE databases best characterized by PDFs including the Gamma family, the generalized extreme value, and exponential distributions. This raises a fundamental question on the ability of such population data to be accurately reflected in clinical tools; tools that largely dominate the research domain and advocate a “*one size fits all*” model (Torres et al., 2016a). Such a model is inadequate as it remains incongruent with empirical data from motions at all levels of nervous systems functioning that our proposed taxonomy defines (Torres, 2011): deliberate-voluntary (Figure 1 and see Torres et al., 2016a); spontaneous-involuntary (explored in this work, Torres and Denisova, 2016; and inevitable-autonomic, Ryu and Torres, 2017).

### The question of medication intake

The present work also demonstrates atypically elevated levels of NSR and randomness in the amplitude fluctuations of the involuntary head micro-movements of female participants with ASD and ASD-related diagnoses (AS and PDDNOS, PDD) in relation to age-matched TD controls, *whether or not they took medication*. That is, even the medication naïve ASD and AS females demonstrated noisy and random involuntary motor signatures. It is our proposition (Brincker and Torres, 2013) that this excess noise from the periphery may compromise kinesthetic feedback, echoing a form of persistently corrupted re-afferent feedback loop. This result is interesting in light of the prior work involving ABIDE I participants (Torres and Denisova, 2016), which had predominantly ASD male participants, but produced results that demonstrated differences according to the quantity of medication intake and medication classes. As such, there seems to be a difference between males and females on the spectrum regarding medication and involuntary head micro-movements. Larger sets involving females only with more detailed medication information (e.g., dosage, class, time on treatment, etc.) will be required to further investigate this hypothesis, nonetheless, the question of medication and mental illness is complex.

These new results are, however, a step forward toward the integration of ordinal *discrete data* from observational inventories with physically driven objective criteria from *continuous data*. In particular, the present criteria are derived directly from biorhythms of the nervous systems—which may mark nervous systems' disorders. Indeed, the 5:1 male to female ratio from observational methods currently employed to diagnose ASD strongly suggests that these observational criteria appear to “miss” the females early in life. In this sense, physical parameters providing objective assessments of somatic-motor measures and other related physiological signatures may boost the early detection rate and help distinguish sub-types of females in the spectrum relative to neuro-typical controls. Building on prior work quantifying differences in patterns of voluntary control that differentiate between males and females with ASD (Torres et al., 2013b) during a decision-making task, the present results demonstrate the ability to detect sex differences by analyzing involuntary head motion extracted from resting state activity during fMRI experiments. Perhaps combining these levels of enquiry we can further refine our understanding between different female subgroups. Specifically, we propose that neurodevelopmental fields dealing with criteria for mental illness, as defined by the DSM and ADOS, may utilize objective metrics grounded on somatic-motor physiology—in a move toward the Precision Psychiatry agenda (Torres et al., 2016a) and the Research Domain Criteria (RDoC) of the NIMH (Insel et al., 2010; Insel, 2014).

Unfortunately, both psychological (ADOS-2/ ADOS-G) and psychiatric (DSM) criteria for the diagnosis of ASD exclude somatic-motor criteria. For instance, the ADOS-2 manual states (author emphasis added):

*“Note that the ADOS-2 was developed for and standardized using populations of children and adults **without significant sensory and motor impairments**. Standardized use of any ADOS-2 module presumes that the individual can walk independently and is free of visual or hearing impairments that could potentially interfere with use of the materials or participation in specific tasks”* (Lord et al., 2012).

While, the DSM-criteria also avoid somatic-motor issues on the grounds that many individuals on the autism spectrum, including infants and young children, are on psychotropic medication which may impact the nervous systems functioning. Indeed, under the DSM-5 (American Psychiatric Association, 2013) section entitled ***“Medication-Induced Movement Disorders and Other Adverse Effects of Medication”***, several disorders are listed as byproducts of adverse effects from psychotropic medication intake. Within this setting, the DSM-5 explicitly states, **“Although these movement disorders are labeled ‘medication induced’, it is often difficult to establish the causal relationship between medication exposure and the development of the movement disorder”**, (DSM-5; medication section, American Psychiatric Association, 2013). While none of this section makes direct reference to developmental disorders like ASD or ADHD that under DSM-5 (but not under DSM-IV) are allowed to be comorbid (American Psychiatric Association, 1994, 2013), such developmental disorders are heavily medicated worldwide (Zito et al., 2003; Chai et al., 2012; Zhang et al., 2013) with uncertain consequences. Future consideration of the impact of medication intake on somatic-motor criteria may help separate involuntary motor issues from those present across the spectrum regardless of medication.

Finally, such results indicate that the ASD and ASD-related female phenotype (i.e., AS, PDDNOS and PDD) can be distinguished according to stochastic signatures of involuntary head micro-movements. Likewise, the ASD male phenotype can be distinguished from the AS and TD controls. However, the age groups in ABIDE start at 6 years of age. These distinctions need to be made within the first couple of years of life before an observational diagnosis or a diagnosis based on parental reports is already in place. By then, the problems are obvious to the naked eye, suggesting they have reached a more steady-state status with a tendency to become harder to readapt once the rates of adaptive change in the nervous systems slow down or plateau.

It is our proposition that perhaps to detect risk for a neurodevelopmental disorder earlier in life, we could begin to combine the types of neuro-motor control related biometrics explained here with patterns of physical growth that are already tracked by pediatricians in the newborn -as we did in a small cohort of 36 babies, some at risk of stunting in neurodevelopment (Torres et al., 2016b). Indeed, female newborn babies are already separable from the male newborn babies according to their patterns of physical growth. This should be particularly important in the nascent nervous systems of the newborn baby, or the rapidly developing nervous system of a young infant. During the pre-cognitive state of the neonate, accelerated rates of change in physical growth are accompanied by rapid neurodevelopment of motor control when typical development is in place (Torres et al., 2016b). Indeed failing to follow this coupled rate of change trajectory reveals stunting in neurodevelopment rather early. As such, objectively tracking physical parameters may help us identify the females with neurodevelopmental issues much earlier than current observational inventories or parental reports allow. The latter are of outmost importance. But if we were to complement them with physical criteria and properly derive and standardize their statistical ranges using normative approaches, more progress on the early detection of risk for neurodevelopmental issues would be ascertained.

## Conclusions

The present methods were adapted to the personalized assessment of nervous systems biorhythms to objectively quantify: (1) the excess involuntary motions present as the person laid down in resting state and was instructed to remain still; (2) the cumulative effects of continuous head motions on the NSR and randomness of this physiological waveform; and to (3) distinguish females across the human spectrum of typical and atypical development resulting in an ASD or AS/PDDNOS diagnosis. Notwithstanding the limitations of the study owing to the need for more females of diverse age groups, more information on medication intake (dosage, classes, time of treatment, etc.), and the issues with the ADOS-based criteria, we demonstrate that it is possible to initiate the path of better defining the ASD female phenotype by employing objective quantitative means and publicly available large data sets. As our bodies are in constant motion (even when seemingly at rest) these methods may be extended to use with wearable sensing technology and cloud updating under the mobile-Health concept, contributing to progress toward a mathematically-driven model of Precision Psychiatry.

## Ethics statement

The datasets generated and/or analyzed during the current study are available in the ABIDE I repository, http://fcon_1000.projects.nitrc.org/indi/abide/abide_I.html.

## Author contributions

ET designed and performed stochastic analyses and wrote paper; SM extracted all head motion data from ABIDE I and ABIDE II; ET, SM, CC, and CW analyzed demographics; ET, SM, and CW analyzed neonates data; SM, CC, and CW edited paper and all authors agreed to the last version of the MS. All authors read and approved the final manuscript. CC and CW independently reproduced all the ADOS-related statistical results and graphs reported in the paper and produced the Supplementary Materials.

### Conflict of interest statement

ET and Rutgers University hold patent pending agreements for the technology used in this manuscript to analyze the data. The other authors declare that the research was conducted in the absence of any commercial or financial relationships that could be construed as a potential conflict of interest.

## Footnotes

^1^https://afni.nimh.nih.gov/pub/dist/doc/program_help/afni_proc.py.html
