# Supplementary material for: Stochastic Signatures of Involuntary Head Micro-movements Can Be Used to Classify Females of ABIDE into Different Subtypes of Neurodevelopmental Disorders
Source: Front Integr Neurosci. 2017 Jun 7;11:10. doi: 10.3389/fnint.2017.00010 (PMC5461345; doi:10.3389/fnint.2017.00010)
Supplement: Supplementary file 1 [file DataSheet1.DOCX]

**Supplementary Material**

As noted, the distribution of each underlying metric of both the ADOS-2 and ADOS-G was utilized to empirically estimate the probability distribution function (PDF) that best fit the data. This was subsequently used to derive the mean and variance. The following provides information regarding this process. The below images provide visualization of the distribution of each ADOS-2 and ADOS-G sub-score for both the ASD and AS groups of both sex (i.e. Male and female). This distribution is subsequently fitted with a range of probability distributions (as illustrated), with the parameters presented in the corresponding tables below. The cumulative probability is further visualized as an inset for reference.

|  |
| --- |
| **Figure 1: ADOS-2 Social Affect sub-scale individual group fitting.** |

|  |
| --- |
| **Figure 2: ADOS-2 Restrictive and Repetitive Behaviors sub-scale individual group fitting.** |

|  |
| --- |
| **Figure 3: ADOS-G Communication sub-scale individual group fitting.** |

|  |
| --- |
| **Figure 4: ADOS-G Social Affect sub-scale individual group fitting.** |

|  |
| --- |
| **Figure 5: ADOS-G Stereotypical behaviors sub-scale individual group fitting.** |

**Table 1**: **Information regarding the sites in ABIDE I and II that contained information in relation to medication intake.**

| ABIDE | SITE NAME | N | COLUMNS |
| --- | --- | --- | --- |
| I | CMU | 27 | AN-AO |
| I | KKI | 55 | AN-AO |
| I | LEUVEN 1 | 29 | AN-AO |
| I | MAX MUN | 57 | AN-AO |
| I | NYU | 184 | AN-AO |
| I | OLIN | 36 | AN-AO |
| I | PITT | 57 | AN-AO |
| I | SBL | 30 | AN-AO |
| I | SDSU | 36 | AN-AO |
| I | STANFORD | 40 | AN-AO |
| I | TRINITY | 49 | AN |
| I | UCLA 1 | 82 | AN-AO |
| I | UCLA 2 | 27 | AN-AO |
| I | UM 1 | 110 | AN-AO |
| I | UM 2 | 35 | AN-AO |
| II | BNI | 58 | BB-BC |
| II | EMC | 54 | BB-BC |
| II | GU | 106 | BB-BC |
| II | IP | 56 | BB-BC |
| II | KKI | 211 | BB-BC |
| II | KUL | 28 | BB-BC |
| II | NYU 1 | 78 | BB-BC |
| II | NYU 2 | 27 | BB-BC |
| II | OHSU | 93 | BB-BC |
| II | ONRC | 59 | BB-BC |
| II | SDSU | 58 | BB-BC |
| II | TCD | 42 | BB-BC |
| II | UCD | 32 | BB-BC |
| II | UCLA | 32 | BB-BC |
| II | USM | 33 | BB-BC |

**Table 2**: **Information regarding the sites in ABIDE I and II that contained information in relation to ADOS-2 and ADOS-G scores.**

| ABIDE | SITE NAME | N | ADOS-2 COLUMNS | | ADOS-G COLUMNS | |
| --- | --- | --- | --- | --- | --- | --- |
| I | CALTECH | 38 | __ | U-X | |  |
| I | CMU | 27 | __ | U-X | |  |
| I | KKI | 55 | Z-AC | U-X | |  |
| I | MAX MUN | 57 | __ | U-X | |  |
| I | NYU | 184 | Z-AC | U-X | |  |
| I | OHSU | 28 | __ | U-X | |  |
| I | OLIN | 36 | Z-AC | U-W | |  |
| I | PITT | 57 | __ | U-X | |  |
| I | SBL | 30 | __ | U-X | |  |
| I | STANFORD | 40 | Z-AC | U-W | |  |
| I | TRINITY | 49 | __ | U | |  |
| I | UCLA 1 | 82 | Z-AC | U-X | |  |
| I | UCLA 2 | 27 | Z-AC | U-X | |  |
| I | UM 1 | 110 | Z-AC | __ | |  |
| I | UM2 | 35 | Z-AC | __ | |  |
| I | USM | 101 | Z-AC | U-X | |  |
| I | YALE | 56 | Z-AC | U-X | |  |
| II | BNI | 58 | AD-AF | Y-AC | |  |
| II | ETH | 37 | __ | Y-AB | |  |
| II | GU | 106 | AD-AG | Y-AB | |  |
| II | IP | 56 | AD-AG | Y-AB | |  |
| II | IU | 40 | __ | Y-AC | |  |
| II | KKI | 211 | AD-AG | Y-AB | |  |
| II | KUL | 28 | __ | Y-AB | |  |
| II | NYU 1 | 78 | AD-AG | Y-AB | |  |
| II | NYU 2 | 27 | AD-AG | Y-AB | |  |
| II | OHSU | 93 | AD-AG | Y-AB | |  |
| II | ONRC | 59 | __ | Y-AB | |  |
| II | SDSU | 58 | AD-AG | __ | |  |
| II | TCD | 42 | __ | Y-AB | |  |
| II | UCD | 32 | AD-AG | Y-AB | |  |
| II | UCLA | 32 | AD-AG | Y-AB | |  |
| II | USM | 33 | __ | Y-AB | |  |

**Table 3. Information regarding distribution fitting across age-normalized (incremental) ADOS-2 scores for female participants with ASD.**

ADOS-2 distribution fitting for **Females with ASD**

| Test Component | Normal | Gamma | Generalized EV | Exponential |
| --- | --- | --- | --- | --- |
| ADOS2  Severity | LogL: 24.2661  µ: 0.4397  σ: 0.0306 | LogL: 27.759  µ: 0.4397  σ: 0.0312 |  |  |
| ADOS2  RRB | LogL: 37.4672  µ: 0.2217  σ: 0.0224 | N/A | LogL: 44.5077  µ: 0.2223  σ: 0.0218 | N/A |
| ADOS2  Social Affect | LogL: 38.7628  µ: 0.2810  σ: 0.0217 | N/A | LogL: 45.3724  µ: 0.2815  σ: 0.0208 | LogL: 20.736  µ: 0.2810  σ: 0.0790 |
| ADOS2  Total | LogL: 52.4193  µ: 0.2704  σ: 0.0152 | LogL: 61.683  µ: 0.2704  σ: 0.1340 |  |  |

LogL: Log Likelihood, µ: mean, σ: variance.

**Table 4. Information regarding distribution fitting across age-normalized (incremental) ADOS-G scores for female participants with ASD.**

ADOS-G distribution fitting for **Females with ASD**

| Test Component | Normal | Gamma | Generalized EV | Exponential |
| --- | --- | --- | --- | --- |
| ADOS-G  Communication | LogL: 30.4617  µ: 0.2441  σ: 0.0263 | N/A | LogL: 39.0888  µ: 0.2442  σ: 0.0281 | LogL: 30.7554  µ: 0.2441  σ: 0.0596 |
| ADOS-G  Social Affect | LogL: 22.2922  µ: 0.3146  σ: 0.0327 | LogL: 29.0029  µ: 0.3146  σ: 0.0349 | LogL: 27.7826  µ: 0.3142  σ: 0.0332 | N/A |
| ADOS-G  Stereotypical Behavior | LogL: 38.6573  µ: 0.1323  σ: 0.0211 | N/A | N/A | LogL: 76.7082  µ: 0.1323  σ: 0.0175 |
| ADOS-G  Total | LogL: 30.2211  µ: 0.2746  σ: 0.0265 | LogL: 38.0697  µ: 0.2746  σ: 0.0277 |  |  |

LogL: Log Likelihood, µ: mean, σ: variance.

**Table 5. Information regarding distribution fitting across age-normalized (incremental) ADOS-2 scores for female participants with AS.**

ADOS-2 distribution fitting for **Females with AS**

| Test Component | Normal | Gamma | Generalized EV | Exponential |
| --- | --- | --- | --- | --- |
| ADOS2  Severity | LogL: 4.0939  µ: 0.5375  σ: 0.0416 | LogL: 4.2732  µ: 0.5375  σ: 0.0432 |  |  |
| ADOS2  RRB | LogL: 5.0613  µ: 0.2970  σ: 0.0379 | N/A | LogL: 6.68719  µ: 0.2964  σ: 0.0367 | LogL: 4.4921  µ: 0.2970  σ: 0.0882 |
| ADOS2  Social Affect | LogL: 13.4348  µ: 0.3270  σ: 0.0171 | LogL: 15.0474  µ: 0.3270  σ: 0.0154 | LogL: 15.2186  µ: 0.3274  σ: 0.0195 | N/A |
| ADOS2  Total | LogL: 15.0512  µ: 0.3184  σ: 0.0146 | LogL: 15.4707  µ: 0.3184  σ: 0.0148 |  |  |

LogL: Log Likelihood, µ: mean, σ: variance.

**Table 6. Information regarding distribution fitting across age-normalized (incremental) ADOS-G scores for female participants with AS.**

ADOS-G distribution fitting for **Females with AS**

| Test Component | Normal | Gamma | Generalized EV | Exponential |
| --- | --- | --- | --- | --- |
| ADOS-G  Communication | LogL: 9.7794  µ: 0.2919  σ: 0.0231 | LogL: 9.4387  µ: 0.2919  σ: 0.0292 | LogL: 10.3307  µ: 0.2915  σ: 0.0214 | N/A |
| ADOS-G  Social Affect | LogL: 7.4916  µ: 0.3779  σ: 0.0291 | LogL: 5.29827  µ: 0.3779  σ: 0.0427 | LogL: 7.78296  µ: 0.3781  σ: 0.0279 | N/A |
| ADOS-G  Stereotypical Behavior | LogL: 6.7742  µ: 0.2242  σ: 0.0313 | N/A | N/A | LogL: 9.9059  µ: 0.2242  σ: 0.0503 |
| ADOS-G  Total | LogL: 7.6735  µ: 0.3500  σ: 0.0286 | LogL: 6.0301  µ: 0.3500  σ: 0.0408 |  |  |

LogL: Log Likelihood, µ: mean, σ: variance.

**Table 7. Information regarding distribution fitting across age-normalized (incremental) ADOS-2 scores for male participants with ASD.**

ADOS-2 distribution fitting for **Males with ASD**

| Test Component | Normal | Gamma | Generalized EV | Exponential |
| --- | --- | --- | --- | --- |
| ADOS2  Severity | LogL: 178.366  µ: 0.3793  σ: 0.0246 | LogL: 190.073  µ: 0.3793  σ: 0.0263 |  |  |
| ADOS2  RRB | LogL: 211.443  µ: 0.2073  σ: 0.0211 | N/A | LogL: 258.758  µ: 0.2072  σ: 0.0214 | LogL: 236.938  µ: 0.2073  σ: 0.0430 |
| ADOS2  Soc Affect | LogL: 267.356  µ: 0.2491  σ: 0.0161 | N/A | LogL: 298.136  µ: 0.249  σ: 0.0158 | LogL: 161.101  µ: 0.2491  σ: 0.0620 |
| ADOS2  Total | LogL: 317.893  µ: 0.2391  σ: 0.0126 | LogL: 362.162  µ: 0.2391  σ: 0.0117 |  |  |

LogL: Log Likelihood, µ: mean, σ: variance.

**Table 8. Information regarding distribution fitting across age-normalized (incremental) ADOS-G scores for male participants with ASD.**

ADOS-G distribution fitting for **Males with ASD**

| Test Component | Normal | Gamma | Generalized EV | Exponential |
| --- | --- | --- | --- | --- |
| ADOS-G  Communication | LogL: 438.383  µ: 0.1707  σ: 0.0132 | N/A | LogL: 496.992  µ: 0.1707  σ: 0.0133 | LogL: 451.322  µ: 0.1707  σ: 0.0292 |
| ADOS-G  Social Affect | LogL: 356.976  µ: 0.2128  σ: 0.0174 | N/A | LogL: 394.656  µ: 0.2127  σ: 0.0176 | LogL: 321.824  µ: 0.2128  σ: 0.0453 |
| ADOS-G  Stereo | LogL: 510.541  µ: 0.0856  σ: 0.0103 | N/A | N/A | LogL: 856.995  µ: 0.0856  σ: 0.0073 |
| ADOS-G  Total | LogL: 489.708  µ: 0.1888  σ: 0.0111 | LogL: 560.398  µ: 0.1888  σ: 0.0108 |  |  |

LogL: Log Likelihood, µ: mean, σ: variance.

**Table 9. Information regarding distribution fitting across age-normalized (incremental) ADOS-2 scores for male participants with AS.**

ADOS-2 distribution fitting for **Males with AS**

| Test Component | Normal | Gamma | Generalized EV | Exponential |
| --- | --- | --- | --- | --- |
| ADOS2  Severity | LogL: 25.0149  µ: 0.4496  σ: 0.0390 | LogL: 24.1685  µ: 0.4496  σ: 0.0461 |  |  |
| ADOS2  RRB | LogL: 43.5739  µ: 0.2580  σ: 0.0287 | N/A | LogL: 57.3615  µ: 0.2579  σ: 0.0285 | LogL: 42.9296  µ: 0.2580  σ: 0.0666 |
| ADOS2  Social Affect | LogL: 55.3384  µ: 0.2898  σ: 0.0236 | LogL: 65.5878  µ: 0.2898  σ: 0.0244 | LogL: 66.1824  µ: 0.2895  σ: 0.0237 | N/A |
| ADOS2  Total | LogL: 68.3579  µ: 0.2807  σ: 0.0191 | LogL: 78.5227  µ: 0.2807  σ: 0.0190 |  |  |

LogL: Log Likelihood, µ: mean, σ: variance.

**Table 10. Information regarding distribution fitting across age-normalized (incremental) ADOS-G scores for male participants with AS.**

ADOS-G distribution fitting for **Males with AS**

| Test Component | Normal | Gamma | Generalized EV | Exponential |
| --- | --- | --- | --- | --- |
| ADOS-G  Communication | LogL: 102.959  µ: 0.1521  σ: 0.0169 | N/A | LogL: 130.577  µ: 0.1532  σ: 0.0204 | LogL: 145.718  µ: 0.1521  σ: 0.0231 |
| ADOS-G  Social Affect | LogL: 76.2777  µ: 0.1947  σ: 0.0234 | N/A | LogL: 100.146  µ: 0.1952  σ: 0.0257 | LogL: 104.953  µ: 0.1947  σ: 0.0379 |
| ADOS-G  Stereotypical Behavior | LogL: 149.919  µ: 0.0807  σ: 0.0096 | N/A | N/A | LogL: 250.318  µ: 0.0807  σ: 0.0065 |
| ADOS-G  Total | LogL: 121.289  µ: 0.1772  σ: 0.0135 | LogL: 154.591  µ: 0.1772  σ: 0.0118 |  |  |

LogL: Log Likelihood, µ: mean, σ: variance.
