# Supplementary material for: Stochastic Signatures of Involuntary Head Micro-movements Can Be Used to Classify Females of ABIDE into Different Subtypes of Neurodevelopmental Disorders
Source: Front Integr Neurosci. 2017 Jun 7;11:10. doi: 10.3389/fnint.2017.00010 (PMC5461345; doi:10.3389/fnint.2017.00010)
Supplement: Supplementary file 2 [file Table1.docx]

# Appendix - Tables

## Table 1

Statistical summary of age-normalized (incremental) ADOS-2 and ADOS-G scores for females are shown with p-values from pairwise comparison using the Rank-sum test. Distribution fitting revealed the Gamma distribution as the best fit in all cases but the incremental ADOS-G social which was best fit by the normal distribution.

ADOS-2 Comparison

| FEMALES Test Type | ASD | AS | p-value |
| --- | --- | --- | --- |
| ADOS2  Severity | Gamma  µ: 0.4397  σ: 0.0312 | Gamma  µ: 0.5375  σ: 0.0432 | 0.0267 * |
| ADOS2  RRB | Generalized Extreme  µ: 0.2223  σ: 0.0218 | Generalized Extreme  µ: 0.2964  σ: 0.0366 | 0.1130 |
| ADOS2  Social Affect | Generalized Extreme  µ: 0.2815  σ: 0.0208 | Generalized Extreme  µ: 0.3274  σ: 0.0195 | 0.0818 |
| ADOS2  Total | Gamma  µ: 0.2704  σ: 0.0134 | Gamma  µ: 0.3184  σ: 0.0148 | 0.0494 * |

ADOS-G Comparison

| FEMALES Test Type | ASD | AS | p-value |
| --- | --- | --- | --- |
| ADOS-G  Communication | Generalized Extreme  µ: 0.2442  σ: 0.0281 | Generalized Extreme  µ: 0.2915  σ: 0.0214 | 0.1332 |
| ADOS-G  Social | Gamma  µ: 0.3146  σ: 0.0349 | Generalized Extreme  µ: 0.3781  σ: 0.0279 | 0.0714 |
| ADOS-G  Stereotypical Behavior | Generalized Extreme  µ: 0.1323  σ: 0.0175 | Exponential  µ: 0.2242  σ: 0.0503 | 0.0208 * |
| ADOS-G  Total | Gamma  µ: 0.2746  σ: 0.0277 | Gamma  µ: 0.3500  σ: 0.0408 | 0.0570 * |

## Table 2

Statistical summary of age-normalized (incremental) ADOS-2 and ADOS-G scores for males are shown with p-values from pairwise comparison using the Rank-sum test. Distribution fitting revealed the Gamma distribution as the best fit in all cases except the incremental ADOS-G social which was best fit by the normal distribution.

ADOS-2 Comparisons

| MALES Test Type | ASD | AS | p-value |
| --- | --- | --- | --- |
| ADOS2  Severity | Gamma  µ: 0.3793  σ: 0.0263 | Gamma  µ: 0.4496  σ: 0.0461 | 2.25e-04 * |
| ADOS2  RRB | Generalized Extreme  µ: 0.2072  σ: 0.0214 | Generalized Extreme  µ: 0.2579  σ: 0.0285 | 0.0016 * |
| ADOS2  Social Affect | Generalized Extreme  µ: 0.2495  σ: 0.0158 | Generalized Extreme  µ: 0.2895  σ: 0.0237 | 0.0113 * |
| ADOS2  Tot | Gamma  µ: 0.2391  σ: 0.0117 | Gamma  µ: 0.2807  σ: 0.0190 | 0.0023 * |

ADOS-G Comparisons

| MALES Test Type | ASD | AS | p-value |
| --- | --- | --- | --- |
| ADOS-G  Communication | Generalized Extreme  µ: 0.1710  σ: 0.0133 | Generalized Extreme  µ: 0.1532  σ: 0.0204 | 0.0079 * |
| ADOS-G  Soc | Generalized Extreme  µ: 0.2127  σ: 0.0176 | Exponential  µ: 0.1947  σ: 0.0379 | 0.0260 * |
| ADOS-G  Stereotypical Behavior | Exponential  µ: 0.0856  σ: 0.0073 | Exponential  µ: 0.0807  σ: 0.0065 | 0.5922 |
| ADOS-G  Total | Gamma  µ: 0.1888  σ: 0.0108 | Gamma  µ: 0.1772  σ: 0.0118 | 0.0595 * |

## Table 3

Gamma first and second moments from normalized head excursions

| Participant Type | Mean | Var |
| --- | --- | --- |
| TD | 0.0074 | 2.42e-05 |
| ASD | 0.0195 | 0.0003 |
| AS | 0.0187 | 0.0002 |

## Table 4

Statistical summary of age-normalized (incremental) ADOS-2 and ADOS-G scores for female and male participants with a diagnosis of ASD are shown with p-values from pairwise comparison using the Rank-sum test. Distribution fitting revealed the Gamma, generalized extreme and exponential distributions as the best fit in all case—using which the mean and variance were empirically estimated.

ADOS-2 Comparisons

| Female - Male  ASD Comparisons | Female ASD | Male ASD | p-value |
| --- | --- | --- | --- |
| ADOS2  Severity | Gamma  µ: 0.4397  σ: 0.0312 | Gamma  µ: 0.3793  σ: 0.0263 | 0.0086* |
| ADOS2  RRB | Generalized Extreme  µ: 0.2223  σ: 0.0218 | Generalized Extreme  µ: 0.2072  σ: 0.0214 | 0.2515 |
| ADOS2  Social Affect | Generalized Extreme  µ: 0.2815  σ: 0.0208 | Generalized Extreme  µ: 0.2495  σ: 0.0158 | 0.0853 |
| ADOS2  Total | Gamma  µ: 0.2704  σ: 0.0134 | Gamma  µ: 0.2391  σ: 0.0117 | 0.0176* |

ADOS-G comparisons

| Female Male Test | Female ASD | Male ASD | p-value |
| --- | --- | --- | --- |
| ADOS-G  Communication | Generalized Extreme  µ: 0.2442  σ: 0.0281 | Generalized Extreme  µ: 0.1707  σ: 0.0133 | 1.15e-04* |
| ADOS-G  Soc | Gamma  µ: 0.3146  σ: 0.0349 | Generalized Extreme  µ: 0.2127  σ: 0.0176 | 1.21e-06* |
| ADOS-G  Stereotypical Behavior | Generalized Extreme  µ: 0.1323  σ: 0.0175 | Exponential  µ: 0.0856  σ: 0.0073 | 0.0198* |
| ADOS-G  Total | Gamma  µ: 0.2746  σ: 0.0277 | Gamma  µ: 0.1888  σ: 0.0108 | 7.55e-06* |

## Table 5

Statistical summary of age-normalized (incremental) ADOS-2 and ADOS-G scores for female and male participants with a diagnosis of AS (Asperger’s syndrome) are shown with p-values from pairwise comparison using the Rank-sum test. Distribution fitting revealed the Gamma, generalized extreme and exponential distributions as the best fit in all case—using which the mean and variance were empirically estimated.

ADOS-2 Comparisons

| Female - Male  AS Comparisons | Female AS | Male AS | p-value |
| --- | --- | --- | --- |
| ADOS2  Severity | Gamma  µ: 0.5375  σ: 0.0432 | Gamma  µ: 0.4496  σ: 0.0461 | 0.0767 |
| ADOS2  RRB | Generalized Extreme  µ: 0.2964  σ: 0.0367 | Generalized Extreme  µ: 0.2579  σ: 0.0285 | 0.3887 |
| ADOS2  Social Affect | Generalized Extreme  µ: 0.3274  σ: 0.0195 | Generalized Extreme  µ: 0.2895  σ: 0.0237 | 0.1863 |
| ADOS2  Total | Gamma  µ: 0.3184  σ: 0.0148 | Gamma  µ: 0.2807  σ: 0.0190 | 0.1444 |

ADOS-G Comparisons

| Female Male Test | Female AS | Male AS | p-value |
| --- | --- | --- | --- |
| ADOS-G  Communication | Generalized Extreme  µ: 0.2915  σ: 0.0214 | Generalized Extreme  µ: 0.1532  σ: 0.0204 | 1.31e-04* |
| ADOS-G  Soc | Generalized Extreme  µ: 0.3781  σ: 0.0279 | Exponential  µ: 0.1947  σ: 0.0379 | 1.88e-05* |
| ADOS-G  Stereotypical Behavior | Generalized Extreme  µ: 0.2242  σ: 0.0503 | Exponential  µ: 0.0807  σ: 0.0065 | 1.07e-04* |
| ADOS-G  Total | Gamma  µ: 0.3500  σ: 0.0408 | Gamma  µ: 0.1772  σ: 0.0118 | 2.64e-05* |
